# Spatial Phylogenetics, Biogeographical Patterns and Conservation Implications of the Endemic Flora of Crete (Aegean, Greece) under Climate Change Scenarios

**DOI:** 10.3390/biology9080199

**Published:** 2020-07-31

**Authors:** Konstantinos Kougioumoutzis, Ioannis P. Kokkoris, Maria Panitsa, Panayiotis Trigas, Arne Strid, Panayotis Dimopoulos

**Affiliations:** 1 Department of Ecology and Systematics, Faculty of Biology, National and Kapodistrian University of Athens, Panepistimiopolis, 15701 Athens, Greece; 2 Division of Plant Biology, Laboratory of Botany, Department of Biology, University of Patras, 26504 Patras, Greece; ipkokkoris@upatras.gr (I.P.K.); mpanitsa@upatras.gr (M.P.); pdimopoulos@upatras.gr (P.D.); 3 Laboratory of Systematic Botany, Department of Crop Science, Agricultural University of Athens, Iera Odos 75, 11855 Athens, Greece; trigas@aua.gr; 4 Bakkevej 6, DK-5853 Ørbæk, Denmark; arne.strid@youmail.dk

**Keywords:** CANAPE, conservation prioritization, ecosystem services, endemism, Mediterranean flora, phylogenetic diversity, phylogenetic endemism, plant diversity, vascular plants

## Abstract

Human-induced biodiversity loss has been accelerating since the industrial revolution. The climate change impacts will severely alter the biodiversity and biogeographical patterns at all scales, leading to biotic homogenization. Due to underfunding, a climate smart, conservation-prioritization scheme is needed to optimize species protection. Spatial phylogenetics enable the identification of endemism centers and provide valuable insights regarding the eco-evolutionary and conservation value, as well as the biogeographical origin of a given area. Many studies exist regarding the conservation prioritization of mainland areas, yet none has assessed how climate change might alter the biodiversity and biogeographical patterns of an island biodiversity hotspot. Thus, we conducted a phylogenetically informed, conservation prioritization study dealing with the effects of climate change on Crete’s plant diversity and biogeographical patterns. Using several macroecological analyses, we identified the current and future endemism centers and assessed the impact of climate change on the biogeographical patterns in Crete. The highlands of Cretan mountains have served as both diversity cradles and museums, due to their stable climate and high topographical heterogeneity, providing important ecosystem services. Historical processes seem to have driven diversification and endemic species distribution in Crete. Due to the changing climate and the subsequent biotic homogenization, Crete’s unique bioregionalization, which strongly reminiscent the spatial configuration of the Pliocene/Pleistocene Cretan paleo-islands, will drastically change. The emergence of the ‘Anthropocene’ era calls for the prioritization of biodiversity-rich areas, serving as mixed-endemism centers, with high overlaps among protected areas and climatic refugia.

## 1. Introduction

Biodiversity loss due to human actions has been accelerating since the industrial revolution [1], with current extinction rates being well above the background extinction rate [2,3], despite the resilience of plant taxa to extinction [4,5]. This is altering biogeographical patterns [6] and leading to biotic homogenization across scales, with geographically widespread species gaining ground and occurring more frequently in disturbed habitats due to land degradation, replacing narrow-ranged, rare species in natural habitats as well [7]. This phenomenon is likely to be exacerbated in the near future, due to synergistic effects of climate and land-use change [8,9].

To face this phenomenon, the Convention on Biological Diversity established the Aichi Targets, with targets 11 and 12 calling for the establishment of a bare minimum regarding the percentage of terrestrial land under protection and the prevention of known threatened species, respectively [10]. Nevertheless, we are lagging behind both those goals and the post-2020 global biodiversity agenda might need to re-evaluate these targets [11]. In the economic depression era [12], the limited conservation funds need to be allocated in a way that optimizes species management, protection and conservation [13], as well as relevant ecosystem services [14]. The conservation prioritization schemes should focus on areas where the intersection between the different facets of biodiversity (i.e., taxonomic, phylogenetic and functional) is high [15] and there is a balance between conservation actions and costs [16]. These biodiversity-rich areas (hotspots) may occur in climatically stable areas [17], constituting macrorefugia [18] (i.e., climatically stable and biodiversity-rich areas), which usually have high irreplaceability values (a measure of the conservation value of a given area [19,20]) and may comprise of centers of endemism [21], which have been proved useful in conservation prioritization [22]. The emerging field of spatial phylogenetics [23] enables the identification of endemism centers, while taking into account several phylogenetic diversity metrics (e.g., [24]), thus providing valuable insights regarding the eco-evolutionary and conservation value, as well as the biogeographical origin of the area under study [15,25,26].

As plant diversity is not evenly distributed across the globe [27], with few countries hosting more than 1000 endemic taxa [28], certain areas have been identified as biodiversity hotspots due to their high species richness and elevated rates of habitat degradation/loss, with at least five of them encompassing islands or archipelagos [29,30]. The Mediterranean Basin, a terrestrial biodiversity hotspot [30,31], is the second largest hotspot in the world [32], comprising of ca. 10,000 islands and islets. Plant endemism reaches up to 18% in the largest Mediterranean islands and up to 40% in their (sub-) alpine zones [33]. The Mediterranean hosts several vulnerable species [34], with climate change projections expected to be more severely expressed on the Mediterranean islands and mountain ranges [33], which may have acted as refugia in the distant past [35]. Consequently, more plant extinctions are anticipated in these areas, even though very few have been documented thus far [36,37], despite intense anthropogenic pressure and plant invasions [38], acting in parallel with climate change.

A critical step towards effective conservation—apart from identifying areas with high (taxonomic and/or phylogenetic) biodiversity and irreplaceability—is the extinction risk assessment of all the (endemic) species occurring in a given area [39]. This constitutes Aichi Target 2 of the Convention on Biological Diversity, which is highly unlikely to be met by the end of 2020 [28], partly due to limited conservation funds, but also due to the Linnean and Wallacean shortfalls [40,41,42]. Greece, one of the most biodiverse and environmentally complex European countries [43], has been no exception to this rule, since most Greek endemic plant taxa have not yet been assessed according to the International Union for Conservation of Nature (IUCN) criteria [28]. Recent advances have addressed the Wallacean shortfall in Greece (e.g., [44]; Flora Hellenica Database (ongoing): 1.2M records). Recently, all the single island endemics (SIEs) of Crete, the hottest endemic biodiversity Mediterranean island regional hotspot, have been preliminary assessed under the IUCN Criteria A and B [45]; a small, yet crucial step towards Aichi Target 2 in Greece. To date, very few studies have applied phylogenetic diversity metrics to macroecological analyses in Greece [46,47], while none has incorporated these metrics in conservation prioritization and/or assessment analyses. Moreover, a handful of species-specific studies have dealt with the impacts of climate change on plant distribution patterns [48,49,50]. Furthermore, even though numerous biogeographical studies have been conducted in Greece and in the Aegean archipelago [51,52,53,54,55,56,57,58,59], none have yet assessed how climate change might alter the current biogeographical configuration, which took millennia to form [60]. The Aegean archipelago is an important component of the Mediterranean region [61], as it comprises more than 8000 islands, located at the intersection of Africa, Europe and Asia. Its high environmental and topographical heterogeneity, as well as its complex palaeogeographical history contribute to its high diversity and endemism, and render it an ideal stage for biodiversity and biogeographical studies [44]. The high endemism of the Aegean archipelago is a result of isolation caused by the intricate geography and orography of the region since the Palaeocene [62], followed by speciation in restricted areas such as islands, mountains and coastal plains. The time is thus ripe for the first phylogenetically informed, conservation prioritization study dealing with the effects of climate change on the plant diversity and biogeographical patterns in Greece.

Our general goal is to investigate how climate change will alter endemism, biogeographical and biodiversity patterns in Crete and assess the conservation implications of those alterations. More specifically, we aim to: (i) identify centers of endemism and the factors leading to their creation, as well as how they will be affected by climate change, (ii) assess the impact of climate change on the biogeographical patterns of Crete and (iii) investigate how climate change induced alterations in species richness, ecological generalism and phylogenetic diversity may affect community composition of Cretan endemic plants. In the end, we will provide a cost-effective, climate-smart and phylogenetically informed conservation strategy framework that could be applied at a regional scale.

## 2. Materials and Methods

### 2.1. Study Area

**Figure 1:**
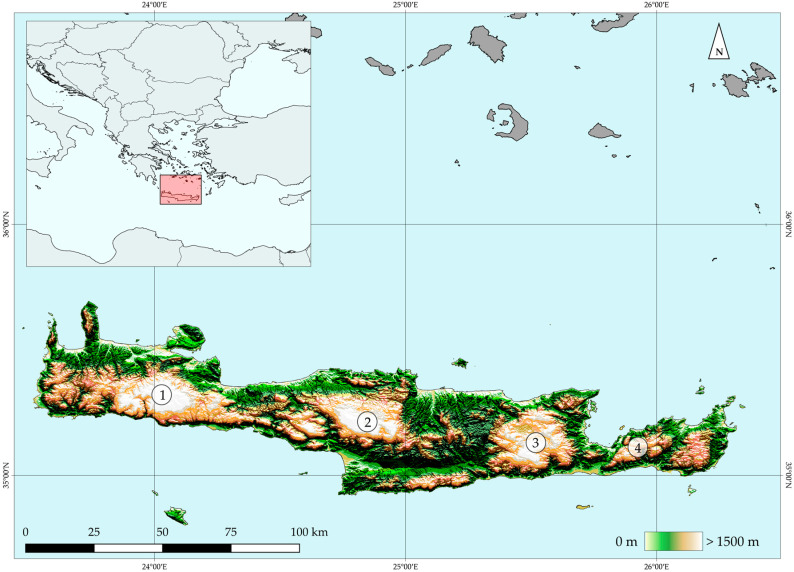
Map depicting Crete in the Mediterranean Sea. Lefka Ori (1), Idi (2), Dikti (3) and Thrypti (4) constitute the main massifs in Crete.

The island of Crete (Greece—Figure 1) has a surface area of 8836 km^2^, thus being one of the largest Mediterranean islands. Crete hosts 2240 native plant species [43,44,63], as well as the most endemic plant species in the Mediterranean Basin [33]: 395 species. Crete hosts 183 single island endemics (SIEs), belonging to 38 families and 104 genera. Regarding the SIE, Caryophyllaceae and Asteraceae constitute the richest families, while *Dianthus* and *Silene* comprise the richest genera. Most of the SIE are characterized as either Critically Endangered or Endangered under the IUCN Criteria A and B [45]. Crete, together with its satellite islands, comprises of a distinct Aegean biogeographical region [52] and is subdivided into nine distinct biogeographical sectors [59]. In Crete, 54 sites are included in the NATURA 2000 protected area network, covering ca. 40% of the island’s extent.

Crete is geologically very diverse—even though limestone is the dominant geological substrate —and belongs to the Hellenic arc [64]. The island was formed as a result of the subduction of the African plate beneath the Aegean microplate [64]. Crete is characterized by a complicated palaeogeographical history [65,66] and has been shaped by two main geological events, namely its isolation from: (i) the Karpathos’ archipelago and the Cyclades 12 Mya and (ii) Peloponnese after the Messinian salinity crisis [67].

Four mountain ranges (Lefka Ori, Idi, Dikti and Thrypti) with 50 peaks that exceed 2000 m a.s.l. (the highest peak is on Mt. Idi and reaches 2456 m a.s.l.), dominate in a W–E axis the topographically complex terrain of Crete (numerous gorges and ravines occur on the island) and strongly shape its climatic gradient, with the lowland plains being warm and dry.

### 2.2. Environmental Data

Current climatic data were obtained from the WorldClim database [68] at a 30 s resolution. We constructed 16 additional climatic variables at the same resolution via the ‘envirem’ 1.1 [69] R package based on the 19 bioclimatic variables from WorldClim for current climate conditions. We selected three global circulation models (GCMs) that are rendered more suitable and realistic for the study area’s future climate based on [70] and two different Intergovernmental Panel on Climate Change scenarios from the representative concentration pathways (RCPs) family: RCP2.6 (mild scenario) and RCP8.5 (severe scenario). Seven soil variables providing predicted values for the surface soil layer at varying depths, were obtained from the SoilGrids database [71]. Elevation data were derived from the CGIAR-CSI data-portal [72] and then aggregated and resampled using ‘raster’ 2.6.7 R package [73] to match the resolution of the other environmental variables. Climate stability for the past 4 My in Crete, as well as the extent and occurrence of climatic refugia were obtained from [45].

### 2.3. Species Occurrence Data

We used the final dataset from [45] for the current and future potential distribution of 172 SIEs, as eleven SIEs are known from less than three locations and could not be modeled in a species distribution modeling framework [45].

Regarding the phylogenetic data, we used both phylogenies from [45] as a sensitivity analysis. The presence of polytomies and/or randomly resolving polytomies do not impact community-level phylogenetic metrics and the latter are significantly correlated with metrics derived from ‘purpose-built’ phylogenies [74,75]. Nevertheless, all subsequent phylogenetic analyses were computed for both trees. We followed the same methodology as [45] did for the estimation of the phylogenetic alpha diversity (the sum of the branch lengths of all the species consisting of a community [76]—see supplementary materials for a thorough explanation of this method).

### 2.4. Biodiversity Analyses

We followed the categorical analyses of neo- and paleo-endemism (CANAPE) protocol for spatial phylogenetic analyses as set out in [23,77]. We carried out all the relevant analyses in Biodiverse version 3.0 [77]. We first calculated phylogenetic endemism [78] and relative phylogenetic endemism [23], which are the basis for the CANAPE. Phylogenetic endemism is the total branch length from the dated phylogenetic tree of the lineages present at a grid cell divided by the range sizes of the respective lineages [23]. Relative phylogenetic endemism is the ratio between phylogenetic endemism measured from the original phylogeny in relation to the phylogenetic endemism estimated from a phylogeny with equally distributed branch lengths [23]. Relative phylogenetic diversity is also a ratio that compares the phylogenetic diversity observed on the actual tree in the numerator to that observed on a comparison tree in the denominator [23]. To make them easily comparable between analyses, the trees in both the numerator and the denominator are scaled such that branch lengths are calculated as a fraction of the total tree length [23]. The comparison tree retains the actual tree topology but makes all branches of equal length [23]. Thus, relative phylogenetic diversity is the phylogenetic diversity measured on the actual tree divided by the phylogenetic diversity measured on the comparison tree, while relative phylogenetic endemism is the phylogenetic endemism measured on the actual tree divided by phylogenetic endemism measured on the comparison tree [23]. We assessed the statistical significance of phylogenetic diversity, phylogenetic endemism, relative phylogenetic diversity and relative phylogenetic endemism by the following [23] approach. We compared the actual phylogenetic endemism and relative phylogenetic endemism values of each grid cell to the 999 values of a null distribution, using the ‘rand_structured’ option in Biodiverse. We estimated *p*-values from a two-tailed distribution to identify areas with higher (>0.975) or lower (<0.025) phylogenetic endemism or relative phylogenetic endemism than the null distribution [23]. Relative phylogenetic endemism and relative phylogenetic diversity are effective tools for the understanding of the primary evolutionary mechanisms shaping biotas, thus enabling robust conservation assessment and prioritization [79].

CANAPE is a two-step procedure discriminating grid cells with significantly high phylogenetic endemism in neo- or paleo-endemism based on species occurrences and the dated phylogenetic tree ([23]—see supplementary materials for a thorough explanation of this method). Using this method, grid cells can be characterized as neo-, paleo- or mixed centers of endemism. Super-endemism areas represent a subdivision of mixed endemism centers with highly significant concentration of both neo- and paleo-endemics. If a point is significantly high in the relative phylogenetic endemism ratio, then it is a center of paleo-endemism [23]. If a point is significantly low in the relative phylogenetic endemism ratio, then it is a center of neo-endemism [23]. Finally, if a point is significantly high in both the numerator and the denominator (taken alone), but not significant for relative phylogenetic endemism, then it is a center of mixed endemism [23]. As CANAPE results might be sensitive to the grid cell size, CANAPE was also carried out for both phylogenetic trees with four different grid cell sizes across the study area. Overall results for the study area are congruent, regardless of the grid resolution.

All analyses were performed using Perl wrapper functions to run Biodiverse in R modified from https://github.com/NunzioKnerr/biodiverse_pipeline.

### 2.5. Spatial Autoregressive Models

We employed spatial autoregressive models with spatially autocorrelated errors as outlined in [80], to test the relationships among phylogenetic endemism and relative phylogenetic endemism with elevation, pH, mean diurnal range and climate stability (these predictors were not correlated; VIF < 2—the multicollinearity assessment was performed with the ‘usdm’ 1.1.18 R package [81]). Spatial autoregressive models with spatially autocorrelated errors circumvent the problem of the non-independence of residuals related to ordinary least squares regression, as they account for spatial autocorrelation in parameter estimation [82]. All variables were standardized [i.e., (value-mean/standard deviation)] to enhance the comparability of parameter estimates. We used correlograms of the residuals of both spatial autoregressive models with spatially autocorrelated errors and generalized linear models to infer the degree of spatial autocorrelation [80], using functions from the ‘spdep’ 1.1.3 R package [83]. We selected the number of neighbors for the spatial autoregressive models with spatially autocorrelated errors so as to minimize the corrected Akaike information criterion (AICc [80]). We then tested models for all combinations of variables and selected the best model (lower AICc).

### 2.6. Future Diversity and Biogeographical Patterns

We derived species composition in each grid cell under current and future climatic conditions by stacking the presences from the individual species models from [45]. We used a grid cell resolution of 1 km to match the resolution of the predictor variables [84].

#### 2.6.1. Changes in Species Richness (ΔSR)

We estimated projected changes in species richness (ΔSR) by subtracting future projected species richness from current species richness. Negative and positive values represent projected species losses and gains, respectively.

#### 2.6.2. Changes in Phylogenetic Diversity (ΔPD)

We estimated the current and future standardized effect size phylogenetic diversity scores as described above and in supplementary materials for each grid cell. Negative and positive changes in phylogenetic diversity (ΔPD) indicate that assemblages are projected to become increasingly clustered or overdispersed, respectively.

#### 2.6.3. Changes in Ecological Generalism (ΔEG)

Niche breadth [85] is a reasonable surrogate for ecological generalism–specialism of species [86] and ranges between 0 and 1, with higher and lower values indicating generalists and specialists, respectively [85]. We derived the mean of the current niche breadth for the species present in each grid cell under current and future climate scenarios and calculated their difference. Negative and positive changes in ecological generalism (ΔEG) indicate assemblages that are predicted to shift their composition towards a greater proportion of specialists and generalists, respectively [87].

#### 2.6.4. Changes in Phylogenetic Beta Diversity (ΔBD)

Phylogenetic beta diversity is built upon two major components: turnover (β_sim_) and nestedness (β_nes_), which may occur between nested or non-nested assemblages [88,89,90]. Phylogenetic beta diversity and its components were computed using the ‘betapart’ 1.3 R package [91]. We focused on β_sim_ as it contributes more than β_nes_ to overall beta diversity among sites [92,93]. We estimated projected changes in phylogenetic beta diversity (ΔBD) by subtracting future projected β_sim_ from current β_sim_. Negative and positive values indicate a trend towards biotic homogenization and heterogeneity, respectively [87].

We assessed whether changes in β_sim_ were associated with ΔSR, ΔPD, ΔEG and elevation by fitting generalized additive models with the ‘mgcv’ 1.8.31 R package [94] in a model selection framework [95] to determine the best models for describing β_sim_ (see supplementary materials for more details regarding this method).

#### 2.6.5. Changes in Biogeographical Patterns

Following the framework of [96], we estimated the current and future bioregionalization of Crete via an unsupervised classification procedure, using two clustering algorithms: k-means and clustering for large applications) based on the generalized dissimilarity modeling data from [45]. We assessed the optimal number of clusters via the silhouette index [97] for each time period. Finally, we quantitatively assessed the similarity of the different bioregionalizations via the V-measure index of spatial association [98,99]. All analyses were performed using functions from the ‘raster’ 3.0.7 [73], ‘cluster’ 2.0.7-1 [100], ‘clusterCrit’ 1.2.8 [101] and ‘sabre’ 0.3.1 [98] R packages.

## 3. Results

### 3.1. Biodiversity Indices

**Figure 2:**
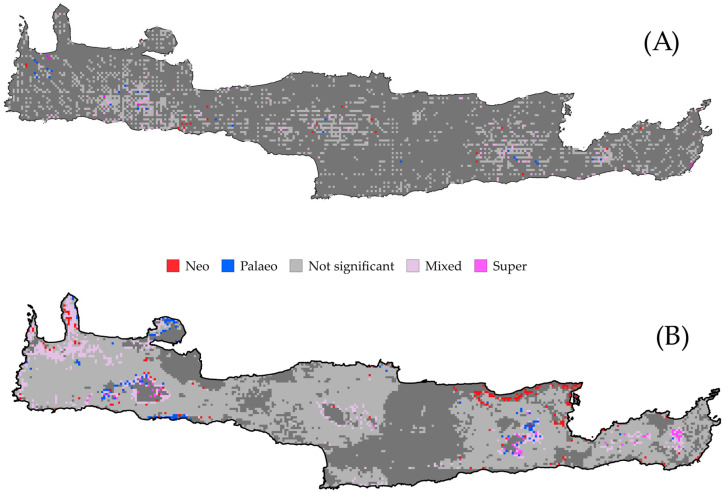
Map of significant phylogenetic endemism (PE) identified by the categorical analysis of neo- and paleo-endemism (CANAPE) analysis for 172 Cretan single island endemics for (**A**) the current time period and (**B**) the CCSM4 global circulation model (GCM) and the representative concentration pathway (RCP) 2.6. Dark grey cells contain no records.

**Figure 3:**
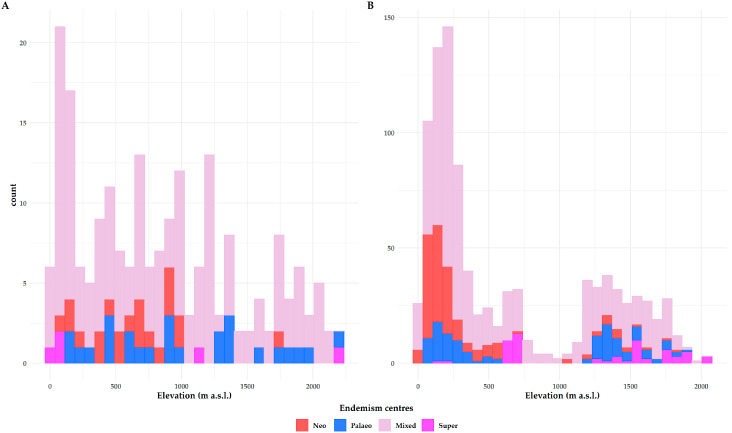
Altitudinal distribution of the different types of endemism centers identified by the CANAPE analysis in Crete for (**A**) the current time period and (**B**) the CCSM4 global circulation model (GCM) and the representative concentration pathway (RCP) 2.6.

The CANAPE analyses revealed that the endemism centers were mainly concentrated within and at the periphery of the four Cretan mountain massifs (Figure 2A). Areas of mixed-endemism were the most common, followed by paleo-, neo- and super-endemism areas (156, 26, 21 and 5, respectively; Figure 2A). Centers of paleo- and mixed-endemism occurred at a significantly higher altitude than not-significant sites (Kruskal–Wallis ANOVA: H = 15.73, df = 4, *p* < 0.01; Table S1; Figure 3). Most paleo-endemism areas occurred in Western Crete and on the mountain massifs of Eastern Crete (Figure 2A). Overall results were congruent, regardless of the grid resolution and the way the phylogenetic tree was constructed (Figures S1 and S2).

The occurrence of the centers of endemism is projected to shift downwards (except for the super-endemism centers) in the future under any GCM/RCP (Figure 2B, Figure 3B and Figure S3, Table S2) and these changes were significantly different for the mixed- and paleo-endemism centers mainly (Kruskal–Wallis ANOVA: H = 344.17, d.f. = 9, *p* < 0.01).

The spatial autoregressive models with spatially autocorrelated errors indicate that elevation was the most important predictor of both phylogenetic endemism and relative phylogenetic endemism (Gelkerke pseudo R-squared (GR^2^) = 16.1% and 5.9%, respectively; Table S3), followed by mean diurnal range and climate stability, respectively.

### 3.2. Future Diversity and Biogeographical Patterns

#### 3.2.1. Changes in ΔEG, ΔPD, ΔSR and ΔBD

**Figure 4:**
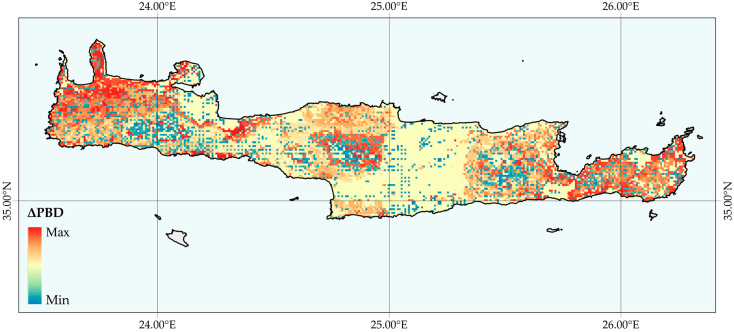
Map of Crete depicting the predicted distribution of areas of biotic homogenization with respect to Cretan single island endemic (SIE) species diversity (projected changes in phylogenetic beta diversity—ΔPBD), according to the CCSM4 2.6 global circulation model/representative concentration pathway compared to the current time period. Blue areas indicate a decrease in beta-diversity (biotic homogenization). Red areas indicate an increase in beta-diversity (biotic heterogenization).

Areas with high SIE β_sim_ were currently concentrated in high altitudes, mainly across the four Cretan mountain massifs (Figure S4a). On the other hand, β_sim_ was relatively low over most of the lowlands and especially in the coastal area of Northern Crete (Figure S4a). β_sim_ was projected to generally increase in Western Crete and in the mid-elevation areas (1000–1500 m a.s.l.—a trend towards biotic heterogeneity) and this pattern was projected to be greatest in a small coastal area of SW Crete (Figure 4). An entirely different pattern emerged mainly in the Cretan highlands, where β_sim_ was predicted to decrease (i.e., a trend towards biotic homogenization—Figure 4 and Figure S4).

The most parsimonious model for all GCMs/RCPs was the full generalized additive model including ΔEG, ΔPD, ΔSR and elevation (Table S4, ΔAIC_C_ < 2; all the other models had ΔAIC_C_ > 2), which explained 71.2%–96.6% of the total variance in β_sim_ (Table S4). Both ΔPD and ΔSR were negatively correlated with the β_sim_ change, while ΔEG was positively correlated with the β_sim_ change (Figures S5–S10). Here we focused on the results obtained for the CCSM4 2.6 GCM/RCP, as it shows the highest similarity with the current conditions (see Section 3.2.2 below) and the patterns and trends did not deviate between the different GCMs and RCPs. The increasing heterogeneity of Western Crete and mid-elevation areas (Figure 4 and Figure S4) was mainly driven by range expansion and local extinction of generalist and specialist species, respectively (Figure S5; see also red areas in Figure S4b,c). Range expansions and local extinctions tended to drive species richness decreases, resulting in phylogenetic clustering (Figure S4d). Hence, future SIE assemblages will tend to be species-poor and comprised of taxa that are more closely related than current assemblages (Figure S5; see also blue areas in Figure S4d). The same processes were largely responsible for the predicted biotic homogenization of the higher elevation areas in Crete, even though these areas were predicted to host more specialist species.

#### 3.2.2. Changes in Biogeographical Patterns

**Figure 5:**
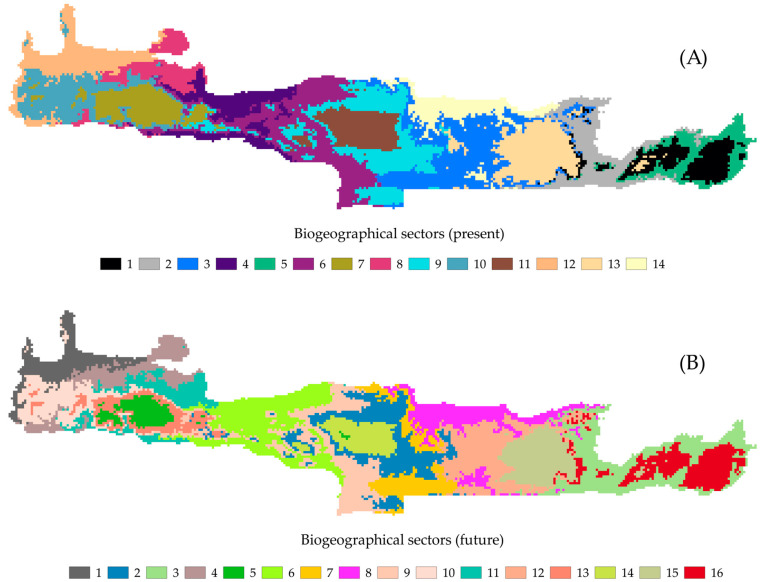
Bioregionalization of Crete for (**A**) the current time period and (**B**) the CCSM4 global circulation model (GCM) and the representative concentration pathway (RCP) 2.6. Each color indicates a different biogeographical sector.

Crete was subdivided into 14 different biogeographical sectors (Figure 5, Figures S11 and S12, Table S5). This biogeographical compartmentalization was predicted to change drastically under any GCM/RCP (Figures S12–S18, Table S5), showing a trend towards biotic homogenization (i.e., decrease in the number of biogeographical sectors—Table S5). Based on the V-measure index, the highest and the lowest similarity was observed for the CCSM4 2.6 and the HadGEM2 8.5 GCM/RCP, respectively (Figure S19, Table S5).

### 3.3. Climatic Refugia and Protected Areas Network Overlap

The overlap between the protected areas network in Crete and endemism centers detected by CANAPE was rather high and ranged between 52% and 65% (Tables S6 and S7; Figure S20a). The overlap between the areas recognized as climatic refugia and endemism centers detected by CANAPE was lower than that reported for the protected areas network and ranged between 0 and 22% (Tables S6 and S7; Figure S20b). Under any GCM/RCP, the predicted overlap for both the protected areas network and the climatic refugia were lower than the one currently observed for most GCMs/RCPs and CANAPE categories (Table S6). The percent overlap based on the recognized climatic refugia of at least the neo-endemism centers was predicted to increase in the future, while the opposite trend was predicted for all the other CANAPE types (Tables S6 and S7).

## 4. Discussion

Crete, one of the largest Mediterranean islands, constitutes the richest regional Mediterranean endemic biodiversity hotspot [33]. It is a speciation center for animal and plant taxa (e.g., [60,102] and references therein), due to its rugged topography, past climatic stability, environmental heterogeneity and long-term isolation, all being crucial factors promoting lineage diversification and persistence [17,103]. Palaeogeographical history, mountain uplift and climate have shaped Crete’s biodiversity and biogeographical patterns (e.g., [59,104,105]). Climate change is expected to modify biodiversity and biogeographical patterns globally [6], with islands and mountain summits exhibiting higher vulnerability to these impacts (e.g., [33,37]). Spatial phylogenetics are a valuable tool for the development of protected areas management schemes, since they reflect spatiotemporal biodiversity patterns (e.g., [106]) and may guide scientific effort towards climate change impact mitigation [107]. Urgent measures to address these effects on biodiversity and to avoid further deterioration are needed, especially concerning the areas of high conservation and evolutionary value that have been highlighted in this study.

### 4.1. Centers of Endemism

In Crete, mixed-endemism centers occurring at a higher altitude than the other types of endemism centers, prevail (Figure 3). It seems that in Crete, montane regions do not act just as diversity cradles, but also as diversity museums—a pattern observed in other parts of the world as well (e.g., [108]) and are considered as important providers of ecosystem services [109]. Most paleo-endemism centers occur in or near ravines/gorges in Western Crete and on the mountain massifs of Eastern Crete (Figure 2A), indicating that these areas may have functioned as biogeographical museums [105,110]. Neo-endemism centers tend to occur in sub-montane altitude in Crete, rather near mixed-endemism centers (Figure 2A and Figure 3A), pinpointing that their recent diversification may have hindered their expansion. In the Pliocene, the Cretan mountain massifs were paleo-islands, reaching 800 m a.s.l., while vast lowland areas were submerged [111]. The SIE altitudinal distribution patterns conform to the mid-domain effect hypothesis [105], peaking at 1500 m a.s.l., due to diversification. Our results lend weight to this notion, since most endemism centers in Crete occur in mid-elevation areas (Table S1). Probably the species present in these paleo-islands migrated and diversified both down- and up-wards, after the sea retracted and Crete acquired its current spatial configuration during the Pleistocene [66]. Most SIE neo-endemics likely diversified in lower altitudes (Figure 2A and Figure 3A; Table S1), probably due to ecological release triggered by low competition [105]. SIE paleo-endemics, which are remnants of an older continental flora [105,112,113], likely persisted in mid- and higher-altitudes (Figure 2A and Figure 3A; Table S1) and at least some of them became high-elevation specialists [105]. Thus, historical processes, such as mountain uplift and isolation due to sea-level oscillations, seem to have driven diversification and endemic species distribution in Crete (e.g., [114]), with this pattern being evident in Cretan endemic animals as well (e.g., [115]) and in other regions of the world [24,107,116,117,118].

In the future, the occurrence of almost all types of endemism centers are projected to shift downwards under any GCM/RCP (Figure 2B, Figure 3B and Figure S3; Table S2), suggesting that montane areas that have served as both diversity cradles and museums for a very long time, will probably become diversity ‘death-zones’ in the near future (see also [45]), as a result of the ‘escalator to extinction’ phenomenon [119]. In many cases, the extent of all types of endemism centers is predicted to significantly shrink (Table S6), due to lineage extinction and/or spatial shift—a phenomenon observed elsewhere as well [120].

Climatic stability and high topographical/environmental heterogeneity may act in conjunction to provide the conditions needed for the simultaneous persistence of paleo-endemics and the diversification of neo-endemics [121]. In this context, altitude—a proxy of environmental heterogeneity [104,105,122,123]—emerged as the most important predictor of PE and RPE in Crete, followed by the mean diurnal range, pH and climate stability. Distinct endemism patterns may be found in sites with phylogenetically distinct assemblages, even if environmental similarity is high, due to phylogenetically conserved niche breadth and dispersal ability [124]. This could be the case in Crete, since SIE’s niche breadth displays a significant, though weak phylogenetic signal [45]. Our results lend weight to the suggestion that the interplay between topographical heterogeneity and climate may be linked with the configuration of centers of paleo- and neo-endemism on mountain massifs [125], a phenomenon also recorded in the Western and Central Mediterranean [107,126], as well as the Neotropics [121]. However, the low GR^2^ for both phylogenetic endemism and relative phylogenetic endemism limits the scope of conclusions we could draw from these results, and so they should be regarded as informative rather than conclusive.

### 4.2. Trends in Biogeographical Patterns

Dispersal limitation, climate differentiation and palaeogeographical configurations have largely shaped the present-day biogeographical division of the Aegean islands ([52] and references therein), with Crete being no exception. The biogeographical affinities of Crete lie with: (i) the Peloponnese to the west, (ii) to the Karpathos’ archipelago to the east and (iii) the South Cyclades to the north (summarized in [52,54,58]), as a result of the turbulent palaeogeographical evolution of the Southern Aegean Sea [53,127]. Crete currently constitutes a distinct Aegean biogeographical region, together with its satellite islands [52]. Nearly three decades ago, [59] divided Crete into nine distinct biogeographical sectors, which they attributed to species-specific altitudinal preferences, based on the then available distribution data. According to our results, Crete is biogeographically subdivided into 14 different sectors (Figure 5 and Figures S11–S18), a result probably of palaeogeographical history and local climate differentiation, since the most important gradient for determining SIE turnover is annual potential evapotranspiration, followed by geographical distance [45] and niche-based processes were found to exert some influence in the distribution of species occurring in Crete [104]. Moreover, Crete’s biogeographical compartmentalization bears a striking resemblance to the spatial configuration of the paleo-islands that were present in the Pliocene/Pleistocene, which correspond roughly with the Cretan mountain massifs [111], a phenomenon observed in several animal taxa as well ([128] and references therein). Migration between those paleo-islands was improbable, even during the Messinian Salinity Crisis, when sea-levels were considerably lower [67], due to unfavorable climatic conditions and the presence of dry steppes and deep canyons [129,130].

This unique biogeographical compartmentalization seems to be at peril, since under any GCM/RCP it will drastically change (Figures S12–S18, Table S5), showing a trend towards biotic homogenization in a W–E axis, along Crete’s altitudinal range. This phenomenon seems to have a greater impact on the SIE of the Cretan highlands, since most of the habitat specialists occurring there, will probably be among the first to become extinct in the next decades [45].

We also predict a trend towards biotic homogenization in the Cretan highlands, whereas an opposite trend towards biotic heterogenization will probably occur in low- and mid-elevation areas in Crete (Figure 4 and Figure S4). This is in line with the thermophilization and phylogenetic diversity decline observed in other regions as well ([131] and references therein). Biotic homogenization is driven by the range expansion of generalists and the consequent local extinction of specialists, resulting in overall lower local species richness and phylogenetic diversity (Figures S5–S10). The aforementioned processes drive biotic heterogenization in Crete, however these heterogeneous assemblages will probably have higher species richness, due to the higher migration rate from the lowlands. The prominent homogenization of the Cretan highlands suggests that climate-driven extinctions of specialists may have greater impact on beta-diversity patterns than the generalists’ range expansion. These regional variation shifts in beta diversity have significant impact on ecoevolutionary processes [132] and could disrupt ecosystem functioning and provisioning of ecosystem services [133].

### 4.3. Conservation Prioritization

There is an urgent need for operational and scalable triage approaches in conservation decision making, due to the accelerating threats to biodiversity, coupled with limited resources ([13,134] and references therein). Conservation triage focuses on prioritizing species, populations or habitats based on biodiversity benefits, recovery potential and costs to achieve a desired goal [135]. Prioritization should be based on species uniqueness and probabilities of extinction, as well as cost of conservation actions [16], even though it is debated whether or not to prioritize paleo- or neo-endemic taxa [134].

A fundamental block for understanding biodiversity patterns and consequently for conservation prioritization is to determine where species diversify (centers of neo-endemism—cradles) and persist (centers of paleo-endemism—museums) over evolutionary time. Irreplaceability is a measure of the conservation value of a given area [19]. An expansion of the existing protected areas [136,137], as is included in the post-2020 biodiversity protection agenda [11] could potentially triple the species’ range under protection, as well as that of phylogenetic/functional units [138]. Many protected areas are also facing increased climate change based risks [139]. In the era of rapid biodiversity decline [140], it is necessary to investigate if different facets of biodiversity are included in existing protected areas [141] and to identify new areas of high evolutionary and conservation value. For these reasons, phylogenetic information, evolutionary history and randomization processes could improve conservation management.

Nearly two decades earlier, the protected areas network in Crete was rendered insufficient to ensure satisfactory representation of the regional plant diversity [142], but as new and detailed data came into light [44], this view has been overturned [45]. The majority of the endemism centers in Crete are within protected areas (Figure S20a; Table S6), a phenomenon observed in mainland Greece [143] and in other regions of the world as well [117]. The recognized climatic refugia in Crete contain mostly mixed- and paleo-endemism centers, while no neo-endemism center is within these refugia (Figure S20b; Table S6). Paleo-endemics are considered extinction-prone and highly threatened in the climate change era [107,120,144,145] and should probably be prioritized in terms of conservation efforts. However, in order to circumvent the problems raised by [134] and building on the results of [45], conservation efforts should focus at areas with high overlaps among protected areas and climatic refugia, characterized simultaneously by high endangered species diversity, as well as serving as mixed-endemism centers. These areas have a relatively small extent, contain both endangered paleo- and neo-endemic species in high numbers and are under protection, while being climatically stable in the past and in the foreseeable future. Thus, they constitute Anthropocene refugia [146] and their management would be cost-effective, in socioeconomic terms. As [120] first suggested, this approach could timely notify conservationists for the impeding loss of different aspects of biodiversity.

### 4.4. Conservation Considerations

Our results are in line with the emergence of the ‘Homogenocene’ era, observed in other regions and organisms as well (e.g., [87,147]). This phenomenon of biotic homogenization could be greatly deteriorated in Crete, if more widespread, more abundant and with different life-strategy taxa (e.g., native non-endemics and aliens) are included in the analyses, as there is mounting evidence of widespread biotic homogenization [147,148], at the taxonomic and phylogenetic scale [149,150]. Alien and invasive species, such as *Agave americana*, *Solanum elaeagnifolium* and *Oxalis pes*-*caprae*, are already occupying vast areas in Crete [151,152], especially in lower to mid-elevation areas, thus already placing immense pressure on (at least some of) the SIE distribution. This phenomenon seems to be affecting more the neo-endemic species, as they usually occur in lower altitudes, as well as those paleo-endemics occurring in the lowland areas of Crete.

In the future, conservationists should address the impact of biotic homogenization on ecosystem services [147], since it is particularly important for policy and decision-making related to land and resource use [153]. To properly assess and predict future projections of landscape fragmentation, changes in demand, use and supply of ecosystem services should be taken into account, using a climate change, management scenario-based approach [154,155].

In Greece, the identification of endemism centers and biodiversity hotspots will aid the mapping and assessment of ecosystems and their services implementation by providing invaluable information, since locating biodiversity hotspots and refugia is a key prerequisite in the national set of mapping and assessment of ecosystems and their services indicators, in order to map and assess biodiversity-related ecosystem services [156].

## 5. Conclusions

This is the first phylogenetically informed, conservation prioritization study taking into consideration the potential impacts of climate change that has ever been conducted in Greece, one of the most species-rich countries of Europe and the Mediterranean Basin. We used Crete, a Mediterranean island regional biodiversity hotspot, as a case study. Even though several climatic refugia exist on Crete and a large part of the island is under efficient protection, urgent measures are needed to halt the biotic homogenization that is under way and preserve the distinct evolutionary heritage present on the high-altitude areas of the study area. Underfunding, taken together with an elevated climate change driven extinction risk, require the application of the cost-effective conservation prioritization scheme that we have developed herein, which could act as a valuable tool to support awareness-raising and decision-making in Greece and at the EU level, in the framework of the EU biodiversity Strategy and the EU Green Deal.

## Supplementary Materials

The following are available online at https://www.mdpi.com/2079-7737/9/8/199/s1, Extended Materials and Methods, **Figure S1:** Categorical analysis of neo- and paleo-endemism (CANAPE) results estimated for coarser geographical scales, based on the phylogeny generated following the framework proposed by [14,15]. (**a**)–(**e**): 0.008, 0.0125, 0.025, 0.05 and 0.1 degrees, respectively. The cells identified as not significant are not depicted. Overall results are congruent, especially for the mixed-endemism patterns, regardless the grid resolution, **Figure S2:** Categorical analysis of neo- and paleo-endemism (CANAPE) results estimated for different geographical scales, based on the phylogeny generated following the framework proposed by [16]. (**a**)–(**e**): 0.008, 0.0125, 0.025, 0.05 and 0.1, and degrees, respectively. Dark grey cells contain no records. Overall results are congruent, especially for the mixed-endemism patterns, regardless of the grid resolution, **Figure S3:** Map of significant phylogenetic endemism (PE) identified by the categorical analysis of neo- and paleo-endemism (CANAPE) analysis for 172 Cretan single island endemics for (**a**) the BCC global circulation model (GCM) and the representative concentration pathway (RCP) 2.6, (**b**) the BCC 8.5 GCM/RCP, (**c**) the CCSM4 2.6 GCM/RCP, (**d**) the CCSM4 8.5 GCM/RCP, (**e**) the HadGEM2 2.6 GCM/RCP and (**f**) the HadGEM2 8.5 GCM/RCP. Dark grey cells contain no records, **Figure S4:** Bivariate maps depicting relative changes in biodiversity measures for Cretan single island endemics between the present and the CCSM4 26 global circulation model/representative concentration pathway. Colors indicate the relative amount of change. The red and blue end of the spectrum indicate reductions and increases, respectively. Each transition in color shading indicates a 10% quantile shift in the value of the variables. (**a**) Beta-diversity, (**b**) species richness (SR), (**c**) average level of ecological generalism (EG) and (**d**) phylogenetic diversity (PD). Yellow areas indicate sites with high current beta-diversity that will continue to have proportionally high beta-diversity. Blue areas indicate sites that beta-diversity is predicted to increase in the future. Green areas indicate sites where beta-diversity will remain largely unchanged. We used a function generated by José Hidasi-Neto to generate the map (http://rfunctions.blogspot.ca/2015/03/bivariate-maps-bivariatemap-function.html), **Figure S5:** Biplots showing the predicted relationships between β_sim_ change and the four environmental predictors included in the best generalized additive model (GAM) for the CCSM4 26 global circulation model/representative concentration pathway. (**a**) Change in species richness (ΔSR). (**b**) Change in phylogenetic diversity (ΔPD). (**c**) Change in elevation. (**d**) Change in the average level of ecological generalism (ΔEG). Fitted lines show the univariate GAMs with 95% confidence interval (dark grey). Rugs on the x-axes show the predictor values and how they are distributed. Labels on the y-axes indicate the smooth functions for the term of interest (ΔSR, ΔPD, ΔEG and elevation) and the estimated degrees of freedom (following the term). Values above and below the horizontal dashed line indicate heterogenization and homogenization, respectively. Values left and right of the vertical dashed line indicate (**a**) species loss and gain, (**b**) PD decrease and increase and (**d**) assemblages composed by specialists and generalists, respectively, **Figure S6:** Biplots showing the predicted relationships between β_sim_ change and the four environmental predictors included in the best generalized additive model (GAM) for the BCC 2.6 GCM/RCP combination. (**a**). Change in species richness (ΔSR). (**b**). Change in phylogenetic diversity (ΔPD). (**c**). Change in elevation. (**d**). Change in the average level of ecological generalism (ΔEG). Fitted lines show the univariate GAMs with 95% confidence interval (dark grey). Rugs on the x-axes show the predictor values and how they are distributed. Labels on the y-axes indicate the smooth functions for the term of interest (ΔSR, ΔPD, ΔEG and elevation) and the estimated degrees of freedom (following the term). Values above and below the horizontal dashed line indicate heterogenization and homogenization, respectively. Values left and right of the vertical dashed line indicate in (**a**) species loss and gain, (**b**) PD decrease and increase and (**d**) assemblages composed by specialists and generalists, respectively, **Figure S7** Biplots showing the predicted relationships between β_sim_ change and the four environmental predictors included in the best generalized additive model (GAM) for the BCC 8.5 GCM/RCP combination. (**a**). Change in species richness (ΔSR). (**b**). Change in phylogenetic diversity (ΔPD). (**c**). Change in elevation. (**d**). Change in the average level of ecological generalism (ΔEG). Fitted lines show the univariate GAMs with 95% confidence interval (dark grey). Rugs on the x-axes show the predictor values and how they are distributed. Labels on the y-axes indicate the smooth functions for the term of interest (ΔSR, ΔPD, ΔEG and elevation) and the estimated degrees of freedom (following the term). Values above and below the horizontal dashed line indicate heterogenization and homogenization, respectively. Values left and right of the vertical dashed line indicate in (**a**) species loss and gain, (**b**) PD decrease and increase and (**d**) assemblages composed by specialists and generalists, respectively, **Figure S8:** Biplots showing the predicted relationships between β_sim_ change and the four environmental predictors included in the best generalized additive model (GAM) for the CCSM4 8.5 GCM/RCP combination. (**a**). Change in species richness (ΔSR). (**b**). Change in phylogenetic diversity (ΔPD). (**c**). Change in elevation. (**d**). Change in the average level of ecological generalism (ΔEG). Fitted lines show the univariate GAMs with 95% confidence interval (dark grey). Rugs on the x-axes show the predictor values and how they are distributed. Labels on the y-axes indicate the smooth functions for the term of interest (ΔSR, ΔPD, ΔEG and elevation) and the estimated degrees of freedom (following the term). Values above and below the horizontal dashed line indicate heterogenization and homogenization, respectively. Values left and right of the vertical dashed line indicate in (**a**) species loss and gain, (**b**) PD decrease and increase and (**d**) assemblages composed by specialists and generalists, respectively, **Figure S9:** Biplots showing the predicted relationships between β_sim_ change and the four environmental predictors included in the best generalized additive model (GAM) for the HadGEM2 2.6 GCM/RCP combination. (**a**). Change in species richness (ΔSR). (**b**). Change in phylogenetic diversity (ΔPD). (**c**). Change in elevation. (**d**). Change in the average level of ecological generalism (ΔEG). Fitted lines show the univariate GAMs with 95% confidence interval (dark grey). Rugs on the x-axes show the predictor values and how they are distributed. Labels on the y-axes indicate the smooth functions for the term of interest (ΔSR, ΔPD, ΔEG and elevation) and the estimated degrees of freedom (following the term). Values above and below the horizontal dashed line indicate heterogenization and homogenization, respectively. Values left and right of the vertical dashed line indicate in (**a**) species loss and gain, (**b**) PD decrease and increase and (**d**) assemblages composed by specialists and generalists, respectively, **Figure S10:** Biplots showing the predicted relationships between β_sim_ change and the four environmental predictors included in the best generalized additive model (GAM) for the HadGEM2 8.5 GCM/RCP combination. (**a**). Change in species richness (ΔSR). (**b**). Change in phylogenetic diversity (ΔPD). (**c**). Change in elevation. (**d**). Change in the average level of ecological generalism (ΔEG). Fitted lines show the univariate GAMs with 95% confidence interval (dark grey). Rugs on the x-axes show the predictor values and how they are distributed. Labels on the y-axes indicate the smooth functions for the term of interest (ΔSR, ΔPD, ΔEG and elevation) and the estimated degrees of freedom (following the term). Values above and below the horizontal dashed line indicate heterogenization and homogenization, respectively. Values left and right of the vertical dashed line indicate in (**a**) species loss and gain, (**b**) PD decrease and increase and (**d**) assemblages composed by specialists and generalists, respectively, **Figure S11:** The values of the Silhouette index for the k-means and the CLARA unsupervised clustering algorithms regarding the optimal number of biogeographical sectors (clusters) currently occurring in Crete, **Figure S12:** Bioregionalization of Crete for (**a**) the current time-period, (**b**) the BCC Global Circulation Model (GCM) and the Representative Concentration Pathway (RCP) 2.6, (**c**) the BCC 8.5 GCM/RCP, (**d**) the CCSM4 2.6 GCM/RCP, (**e**) the CCSM4 8.5 GCM/RCP, (**f**) the HadGEM2 2.6 GCM/RCP and (**g**) the HadGEM2 8.5 GCM/RCP. Each colour indicates a different biogeographical sector, **Figure S13:** The values of the Silhouette index for the k-means and the CLARA unsupervised clustering algorithms regarding the optimal number of biogeographical sectors (clusters) predicted to occur for the BCC 2.6 GCM/RCP combination in Crete, **Figure S14:** The values of the Silhouette index for the k-means and the CLARA unsupervised clustering algorithms regarding the optimal number of biogeographical sectors (clusters) predicted to occur for the BCC 8.5 GCM/RCP combination in Crete, **Figure S15:** The values of the Silhouette index for the k-means and the CLARA unsupervised clustering algorithms regarding the optimal number of biogeographical sectors (clusters) predicted to occur for the CCSM4 2.6 GCM/RCP combination in Crete, **Figure S16:** The values of the Silhouette index for the k-means and the CLARA unsupervised clustering algorithms regarding the optimal number of biogeographical sectors (clusters) predicted to occur for the CCSM4 8.5 GCM/RCP combination in Crete, **Figure S17:** The values of the Silhouette index for the k-means and the CLARA unsupervised clustering algorithms regarding the optimal number of biogeographical sectors (clusters) predicted to occur for the HadGEM2 2.6 GCM/RCP combination in Crete, **Figure S18:** The values of the Silhouette index for the k-means and the CLARA unsupervised clustering algorithms regarding the optimal number of biogeographical sectors (clusters) predicted to occur for the HadGEM2 8.5 GCM/RCP combination in Crete, **Figure S19:** Similarity regarding Crete’s bioregionalization schema between the present and each Global Circulation Model (GCM) and Representative Concentration Pathway (RCP), based on the V-measure index, **Figure S20:** (**a**) Map of the protected areas (PA) network in Crete overlaid onto the Categorical Analysis of Neo- and Paleo-Endemism (CANAPE) results, (**b**) Map of the recognised climate refugia in Crete overlaid onto the CANAPE results. SCI: Sites of Community Importance. Dark grey cells contain no records, **Table S1**: Summary statistics regarding altitude (m a.s.l.) for the different types of endemism centers as well as for the not-significant sites. NS: not-significant. SD: standard deviation, **Table S2:** Median altitude for the different types of endemism centers for the present, as well as for all global circulation models (GCMs) and representative concentration pathways (RCPs) included in the analyses. * denotes a *p*-value < 0.001 in the Kruskal–Wallis ANOVA, **Table S3:** Best spatial autoregressive error models (SAR_err_) for the relationships among phylogenetic endemism (PE), relative phylogenetic endemism (RPE) and the predictor variables. GR^2^: Gelkerke pseudo R-squared. AICc: Akaike information criterion corrected for small samples. Asterisks denote: * *p* < 0.05, ** *p* < 0.01, *** *p* < 0.001. Alt: Altitude. CS: Climate stability. MDR: Mean diurnal range, **Table S4:** Results for BCC 2.6 GCM/RCP combination of the best (i.e., full) generalized additive model relating change in SIE_C_ beta-diversity to change in species richness (ΔSR), average level of ecological generalism (ΔEG) and phylogenetic diversity (ΔPD), as well as elevation. Variables were standardized (i.e., (value-mean/standard deviation)) prior to analysis. AIC: Akaike Information Criterion. AICc: Akaike Information criterion corrected for small samples. df: Degrees of freedom. F: F-values. logLik: log-likelihood. R^2^_adj_: adjusted R^2^_._ The full model was the only model with ΔAICc < 2. Asterisks denote: * *p* < 0.05, ** *p* < 0.01, *** *p* < 0.001, **Table S5:** Number of biogeographical sectors (BR), silhouette index (SI) for the k-means and clustering for large applications (CLARA) unsupervised clustering algorithms and the V-measure index for the present and every global circulation model (GCM) and representative concentration pathway (RCP) combination, **Table S6:** Percent overlap (%) between the protected areas (PA) network in Crete, the climate refugia (CR) recognized in Crete and the endemism centers detected by the categorical analyses of neo- and paleo-endemism (CANAPE). GCM: Global Circulation Model. RCP: Representative Concentration Pathway. The extent (in km^2^) of each CANAPE category for every GCM/RCP combination is also presented, **Table S7:** Median percent overlap (%) between the protected areas (PA) network in Crete, the climate refugia (CR) recognized in Crete and the endemism centers detected by the categorical analyses of neo- and paleo-endemism (CANAPE) for the present, as well as for the future climate conditions (averaged for all global circulation models and representative concentration pathways).
