# Supplementary material for: Spatial Phylogenetics, Biogeographical Patterns and Conservation Implications of the Endemic Flora of Crete (Aegean, Greece) under Climate Change Scenarios
Source: Biology (Basel). 2020 Jul 31;9(8):199. doi: 10.3390/biology9080199 (PMC7463760; doi:10.3390/biology9080199)
Supplement: Supplementary file 1 [file biology-09-00199-s001.pdf]

Spatial Phylogenetics, Biogeographical Patterns and Conservation Implications of the Endemic Flora of Crete (Aegean, Greece) under Climate Change Scenarios

1. Supplementary Materials and Methods.

1.1. Phylogenetic Diversity

We estimated for each grid cell the phylogenetic alpha diversity (PD [1]) of the species inhabiting each of the grid cells with the 'picante' 1.6-2 R package [2] and the standardized effect size scores with 'PhyloMeasures' 2.1 R package [3]. We tested for non-random patterns in PD by estimating their standardized effect size (SES) scores as:

$$SES = \frac{X_{obs} - mean(X_{null})}{s.d.(X_{null})} \qquad \text{(Equation 1)}$$

where X_{obs} is the observed score within each grid cell and mean (X_{NULL}) and standard deviation (X_{NULL}) are the mean and standard deviation of a null distribution of scores generated by shuffling taxa labels of the grid cell-by-species matrix 999 times. We assessed the statistical significance of the SES scores by calculating two-tailed p-values (quantiles) as:

$$\text{p-values} = \frac{rank_{obs}}{runs+1} \qquad \text{(Equation 2)}$$

where $rank_{obs}$ is the rank of the observed scores compared with those of their null distributions, and runs is the number of randomizations [2,3]. SES scores with $p < 0.05$ and $p > 0.95$ were considered as significantly lower and higher than expected for a given PD value, respectively. Positive SES values indicate phylogenetic overdispersion, whereas negative SES values indicate phylogenetic clustering. The greater sensitivity of SES to more terminal structure makes it better suited to explore assembly processes working at finer temporal and spatial scales [4].

1.2. Biodiversity Analyses

We followed the CANAPE protocol for spatial phylogenetics analyses as set out in [5,6]. We carried out all the relevant analyses in Biodiverse version 3.0 [5]. We first calculated phylogenetic endemism (PE [7]) and relative phylogenetic endemism (RPE [6]). PE is the total branch length from the dated phylogenetic tree of the lineages present at a grid cell divided by the range sizes of the respective lineages. RPE is the ratio between PE measured from the original phylogeny in relation to the PE estimated from a phylogeny with equally distributed branch lengths (see [6] for more details). Relative phylogenetic diversity (RPD) is also a ratio that compares the phylogenetic diversity (PD) observed on the actual tree in the numerator to that observed on a comparison tree in the denominator. To make them easily comparable between analyses, the trees in both the numerator and the denominator are scaled such that branch lengths are calculated as a fraction of the total tree length. The comparison tree retains the actual tree topology but makes all branches of equal length. Thus, RPD is PD measured on the actual tree divided by PD measured on the comparison tree, while RPE is PE measured on the actual tree divided by PE measured on the comparison tree [6]. RPE is the basis for the categorical analyses of neo- and paleo-endemism (CANAPE).

1.2.1. Randomization Tests

We assessed the statistical significance of PD, PE, RPD and RPE by the following [6] approach. We compared the actual PE and RPE values of each grid cells to the 999 values of a null distribution, using the 'rand_structured' option in Biodiverse. In this model, species occurrences in grid cells are randomly reassigned to grid cells without replacement, thus keeping constant both the total number of grid cells for each species and the species richness of each grid cell. We ran 999 randomizations, calculating PD, PE, RPD and RPE for each run. These values formed a null distribution for each grid cell for use in non-parametric tests of the significance of observed values. We estimated *p*-values from a two-tailed distribution of values to identify areas with higher (>0.975) or lower (<0.025) PE or RPE than the null distribution [6].

1.2.2. CANAPE

CANAPE is a two-step procedure discriminating grid cells with significantly high PE in neo- or paleo-endemism based on species occurrences and the dated phylogenetic tree [6].

First, to determine whether a site is a center of significantly high endemism, a grid cell needs to be significantly high (one-tailed test, $\alpha = 0.05$) in the numerator of RPE, the denominator or both.

If (and only if) grid cells pass one of those tests, then they are divided into four meaningful, non-overlapping categories of centers of endemism [6]. If a point is significantly high in the RPE ratio (two-tailed test, $\alpha = 0.05$), then it is a center of paleo-endemism (contains significantly more endemic species on long branches). If a point is significantly low in the RPE ratio (two-tailed test, $\alpha = 0.05$), then it is a center of neo-endemism (contains significantly more endemic species on short branches). If it is significantly high in both the numerator and the denominator (taken alone), but not significant for RPE, then it is a center of mixed endemism. Mixed endemism can be interpreted as a center of endemism having a mix of rare long and rare short branches, so not significantly dominated by either paleo-endemism or neo-endemism. The mixed endemism areas are further subdivided: those grid cells that are significantly high in both the numerator and the denominator at the $\alpha = 0.01$ level are termed super-endemic sites (i.e., highly significant concentration of endemic long and short branches [6]).

All analyses were performed using Perl wrapper functions to run Biodiverse in R modified from https://github.com/NunzioKnerr/biodiverse_pipeline.

1.3. Future Diversity Patterns [Changes in Phylogenetic Beta Diversity (ΔBD)]

From the initial set of 44 predictors, only four were not highly correlated (Spearman rank correlation < 0.7 and VIF < 2 [8]). Multicollinearity assessment was performed with the 'usdm' 1.1.18 package [9]. All variables were standardized [i.e., (value-mean/standard deviation)] to help ensure model convergence and enhance comparability of parameter estimates. Model selection was based on Akaike's information criterion corrected for small sample sizes (AICc). We used the dredge function in the 'MuMIn' 1.15.6 package [10] to run a complete set of models with all possible combinations of the predictor variables and to identify the set of 'best models' according to the widely accepted criterion for different AICc values: ΔAICc < 2 (all models with ΔAICc < 2 are considered as equally parsimonious and as having relatively similar levels of support [11]). If more than one model had ΔAICc < 2, we calculated the relative importance of each variable as the sum of AICc weights (Akaike weights—wAICc) for the models in which the variable was included ([11]; see also [12]). Akaike weights are directly interpreted in terms of each model's probability of being the best supported for explaining the data [11,12]. Finally, we calculated the normalized root mean square error (RMSE) for each set of the 'best' models with the 'sjstats' 0.11.2 R package [13].

2. Supplementary tables and figures

Table S1. Summary statistics regarding altitude (m a.s.l.) for the different types of endemism centres as well as for the not-significant sites. NS: not-significant. SD: standard deviation.

Type	Median	SD	Min	Max
Mixed	811	628	17.1	2149
Neo	652	373	107	1741
Paleo	930	602	166	2215
Super	82	966	32.5	2199
NS	562	515	6.25	2259

Table S2. Median altitude for the different types of endemism centres for the present, as well as for all Global Circulation Models (GCMs) and Representative Concentration Pathways (RCPs) included in the analyses. * denotes a *p*-value < 0.001 in the Kruskal-Wallis ANOVA.

GCM/RCP	Mixed	Neo	Paleo	Super
Current	811	652	930	82
BCC 2.6	191 *	327	108 *	156
BCC 8.5	332 *	562	299 *	239
CCSM4 2.6	307 *	154 *	547	1458
CCSM4 8.5	493 *	448	276 *	344
HadGEM2 2.6	402 *	160 *	161 *	197
HadGEM2 8.5	395	78 *	549	284

Table S3. Best spatial autoregressive error models (SAR_{err}) for the relationships among phylogenetic endemism (PE), relative phylogenetic endemism (RPE) and the predictor variables. GR^2: Gelkerke pseudo R-squared. AICc: Akaike Information criterion corrected for small samples. Asterisks denote: * $p < 0.05$, ** $p < 0.01$, *** $p < 0.001$. Alt: Altitude. CS: Climate stability. MDR: Mean diurnal range.

Response	Predictor	Coefficients	GR^2	AICc
PE	Alt	1.44 ***	16.1	11612.6
	MDR	0.44 **		
	pH	0.40 **		
	CS	0.33 **		
RPE	Alt	1.01 *	5.9	4455.3
	MDR	−0.02		
	pH	−0.03		
	CS	0.03		

Table S4. Results for BCC 2.6 GCM/RCP combination of the best (i.e., full) generalized additive model relating change in SIE beta-diversity to change in species richness (ΔSR), average level of ecological generalism (ΔEG) and phylogenetic diversity (ΔPD), as well as elevation. Variables were standardized [i.e., (value-mean/standard deviation)] prior to analysis. AIC: Akaike Information Criterion. AICc: Akaike Information criterion corrected for small samples. df: Degrees of freedom. F: F-values. logLik: log-likehood. R^2_{adj}: adjusted R^2. The full model was the only model with ΔAICc < 2. Asterisks denote: * $p < 0.05$, ** $p < 0.01$, *** $p < 0.001$.

Model	Predictors	Df	AIC	AICc	LogLik	$R^2_{adj.}$	Deviance (%)	F	*p*
BCC 2.6	-	88.4	20067	20068	−9945	0.710	71.2	-	-
	ΔSR							33.9	***
	ΔPD							2.7	***
	ΔEG							515.5	***
	Elevation							21.2	***
BCC 8.5	-	102	1817	1819	−806	0.933	93.3	-	-
	ΔSR							77.4	***
	ΔPD							30.2	***
	ΔEG							2450.1	***
	Elevation							22.1	***
CCSM4 2.6	-	82.8	11866	11867	−5850	0.850	85.1	-	-
	ΔSR							36.4	***
	ΔPD							22.4	***
	ΔEG							1634.8	***
	Elevation							19.8	***
CCSM4 8.5	-	102	1817	1819	−806	0.933	93.3	-	-
	ΔSR							77.4	***
	ΔPD							30.2	***
	ΔEG							2450.1	***
	Elevation							22.1	***
HadGEM2 2.6	-	82.7	8966	8967				-	-
	ΔSR							85.3	***
	ΔPD							4.05	***
	ΔEG							1695.0	***
	Elevation							93.9	***
HadGEM2 8.5	-	91.7	−6566	−6564	3375	0.966	96.6	-	-
	ΔSR							195.9	***
	ΔPD							23.1	***
	ΔEG							5620.5	***
	Elevation							2.5	***

Table S5. Number of biogeographical sectors (BR), Silhouette index (SI) for the k-means and Clustering for Large Applications (CLARA) unsupervised clustering algorithms and the V-measure index for the present and every Global Circulation Model (GCM) and Representative Concentration Pathway (RCP) combination.

GCM/RCP	BR	SI k-Means	SI CLARA	*V*-Measure
Present	14	0.430	0.410	1.000
BCC 2.6	7	0.414	0.392	0.714
BCC 8.5	4	0.475	0.432	0.533
CCSM4 2.6	16	0.421	0.400	0.769
CCSM4 8.5	12	0.499	0.412	0.503
HADGEM2 2.6	9	0.429	0.377	0.683
HADGEM2 8.5	2	0.431	0.430	0.338

Table S6. Percent overlap (%) between the protected areas (PA) network in Crete, the climate refugia (CR) recognised in Crete and the endemism centres detected by the Categorical Analyses of Neo- and Paleo-Endemism (CANAPE). GCM: Global Circulation Model. RCP: Representative Concentration Pathway. The extent (in km^2) of each CANAPE category for every GCM/RCP combination is also presented.

Type	GCM/RCP	Mixed	Neo	Paleo	Super
PA	Present	63	52	65	60
	BCC 2.6	23	45	44	22
	BCC 8.5	18	52	30	43
	CCSM4 2.6	47	28	65	61
	CCSM4 8.5	23	20	13	0
	HadGEM2 2.6	45	38	42	40
	HadGEM2 8.5	22	0	0	0
CR	Present	22	0	4.9	0.7
	BCC 2.6	6.7	13.6	3.1	0
	BCC 8.5	0.01	14.3	1.6	0
	CCSM4 2.6	9.4	4.5	23.2	29.5
	CCSM4 8.5	0	0	0	0
	HadGEM2 2.6	13.2	6.9	7.5	20.8
	HadGEM2 8.5	0	0	0	0
Extent	Present	109.4	14.7	18.2	3.5
	BCC 2.6	555	15	22	26
	BCC 8.5	133	15	89	15
	CCSM4 2.6	446	123	85	43
	CCSM4 8.5	61.8	3.5	5.6	42
	HadGEM2 2.6	219	20	47	34
	HadGEM2 8.5	6.6	2.8	0.7	2.1

Table S7. Median percent overlap (%) between the protected areas (PA) network in Crete, the climate refugia (CR) recognised in Crete and the endemism centres detected by the Categorical Analyses of Neo- and Paleo-Endemism (CANAPE) for the present, as well as for the future climate conditions (averaged for all Global Circulation Models and Representative Concentration Pathways).

Type	Future State		Current State	
	PA	CR	PA	CR
Mixed	23.00	3.36	63.00	22.00
Neo	33.00	5.70	52.00	0.00
Paleo	36.00	2.35	65.00	4.90
Super	31.00	0.00	60.00	0.70

Supplementary Figures

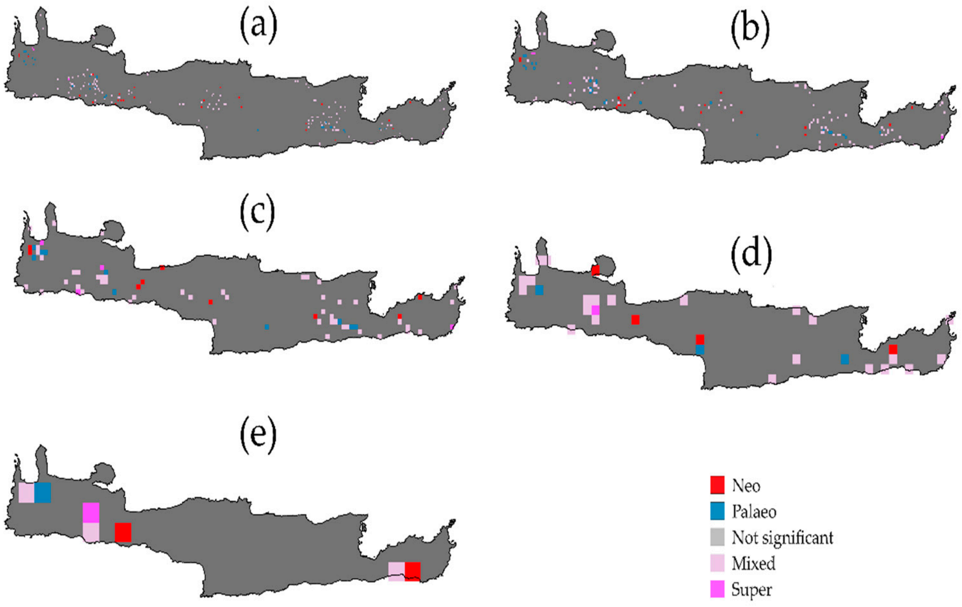

Figure S1. Categorical Analysis of Neo- and Paleo-Endemism (CANAPE) results estimated for coarser geographical scales , based on the phylogeny generated following the framework proposed by [14,15]. (**a**)–(**e**): 0.008, 0.0125, 0.025, 0.05 and 0.1 degrees, respectively. The cells identified as not significant are not depicted. Overall results are congruent, especially for the mixed-endemism patterns, regardless the grid resolution.

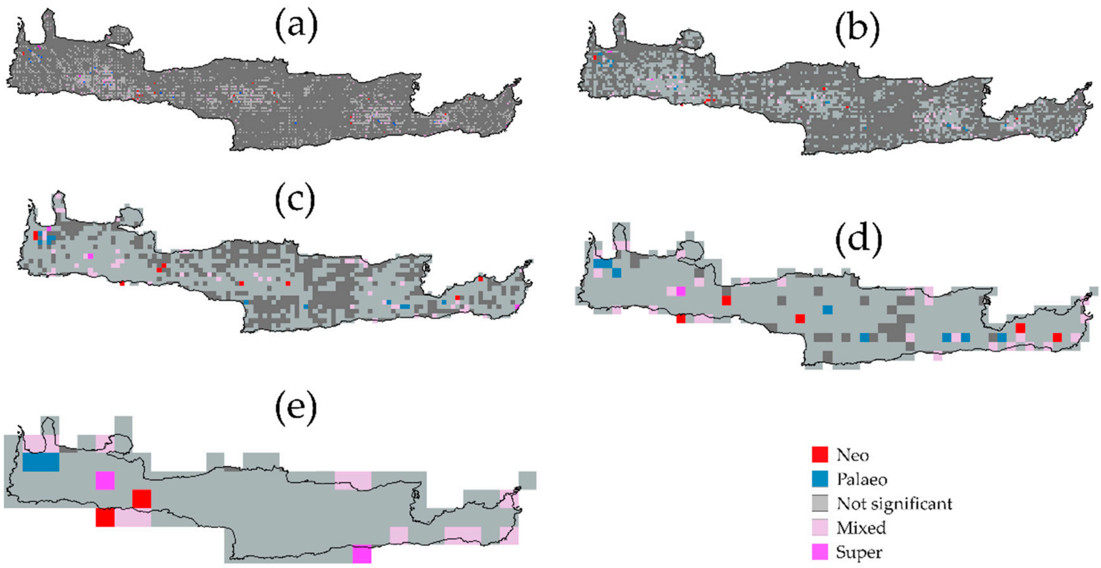

Figure S2. Categorical Analysis of Neo- and Paleo-Endemism (CANAPE) results estimated for different geographical scales, based on the phylogeny generated following the framework proposed by [16]. (**a**)–(**e**): 0.008, 0.0125, 0.025, 0.05 and 0.1, and degrees, respectively. Dark grey cells contain no records. Overall results are congruent, especially for the mixed-endemism patterns, regardless the grid resolution.

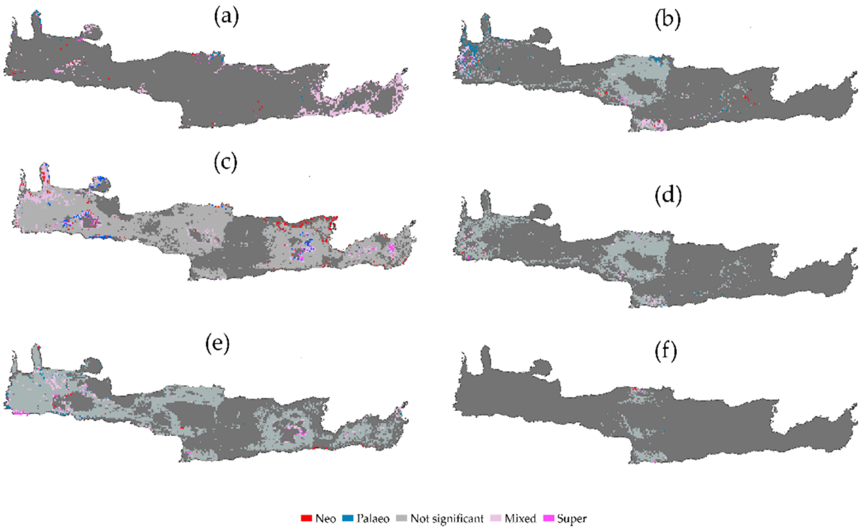

Figure S3. Map of significant phylogenetic endemism (PE) identified by the Categorical Analysis of Neo- and Paleo-Endemism (CANAPE) analysis for 172 Cretan Single Island Endemics for (**a**) the BCC Global Circulation Model (GCM) and the Representative Concentration Pathway (RCP) 2.6, (**b**) the BCC 8.5 GCM/RCP, (**c**) the CCSM4 2.6 GCM/RCP, (**d**) the CCSM4 8.5 GCM/RCP, (**e**) the HadGEM2 2.6 GCM/RCP and (**f**) the HadGEM2 8.5 GCM/RCP. Dark grey cells contain no records.

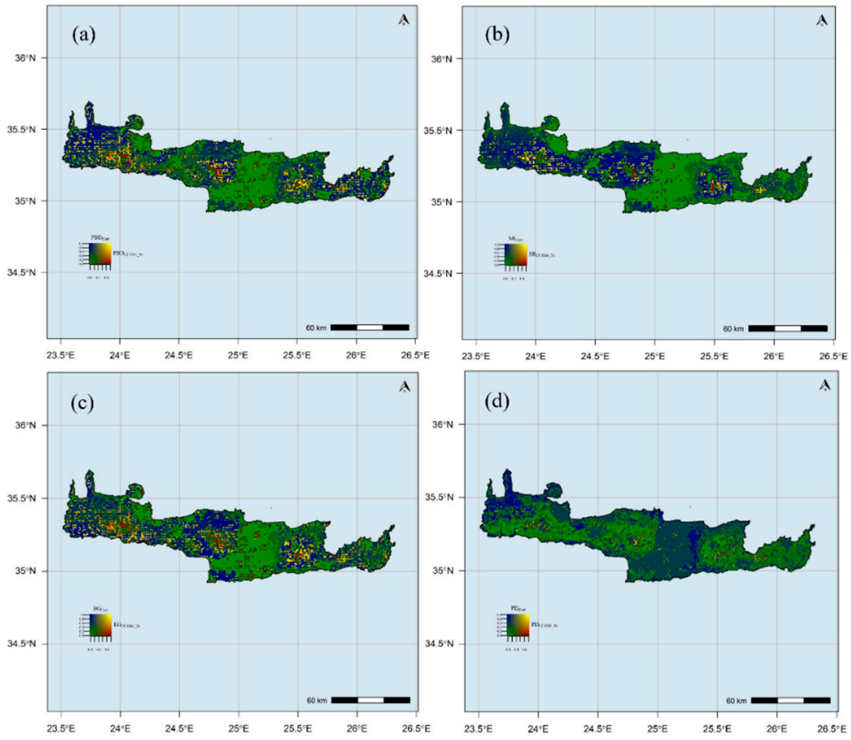

Figure S4. Bivariate maps depicting relative changes in biodiversity measures for Cretan single island endemics between the present and the CCSM4 26 Global Circulation Model/Representative Concentration Pathway. Colours indicate the relative amount of change. The red and blue end of the spectrum indicate reductions and increases, respectively. Each transition in colour shading indicates a 10% quantile shift in the value of the variables. (**a**) beta-diversity, (**b**) species richness (SR), (**c**) average level of ecological generalism (EG), (**d**) phylogenetic diversity (PD). Yellow areas indicate sites with high current beta-diversity that will continue to have proportionally high beta-diversity. Blue areas indicate sites that beta-diversity is predicted to increase in the future. Green areas indicate sites where beta-diversity will remain largely unchanged. We used a function generated by José

Hidasi-Neto to generate the map (http://rfunctions.blogspot.ca/2015/03/bivariate-maps-bivariatemap-function.html).

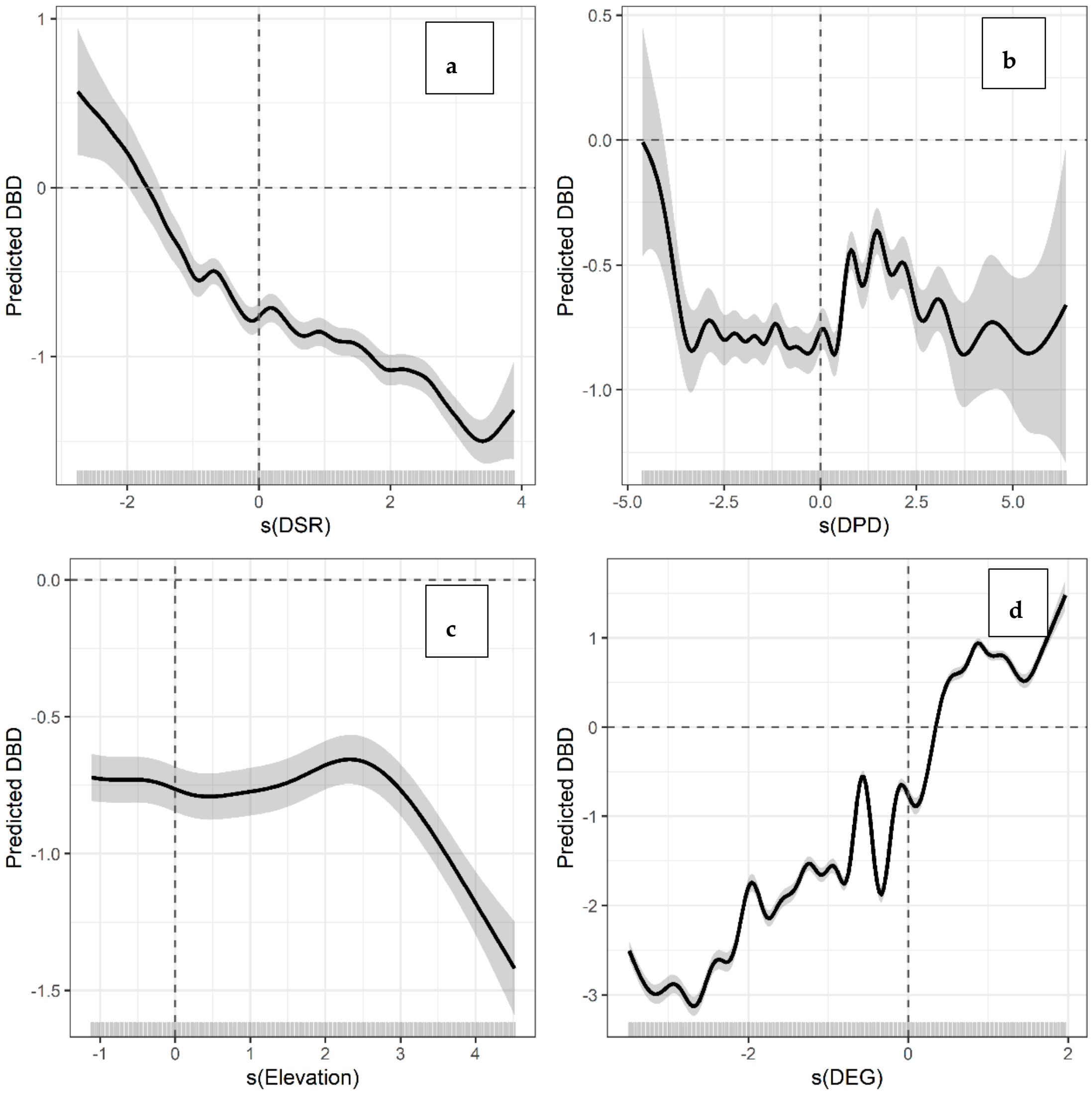

Figure S5. Biplots showing the predicted relationships between β_{sim} change and the four environmental predictors included in the best generalized additive model (GAM) for the CCSM4 26 Global Circulation Model/Representative Concentration Pathway. (**a**) Change in species richness (ΔSR). (**b**) Change in phylogenetic diversity (ΔPD). (**c**) Change in elevation. (**d**) Change in the average level of ecological generalism (ΔEG). Fitted lines show the univariate GAMs with 95% confidence interval (dark grey). Rugs on the x-axes show the predictor values and how they are distributed. Labels on the y-axes indicate the smooth functions for the term of interest (ΔSR, ΔPD, ΔEG and elevation) and the estimated degrees of freedom (following the term). Values above and below the horizontal dashed line indicate heterogenization and homogenization, respectively. Values left and right of the vertical dashed line indicate (**a**) species loss and gain, (**b**) PD decrease and increase and (**d**) assemblages composed by specialists and generalists, respectively.

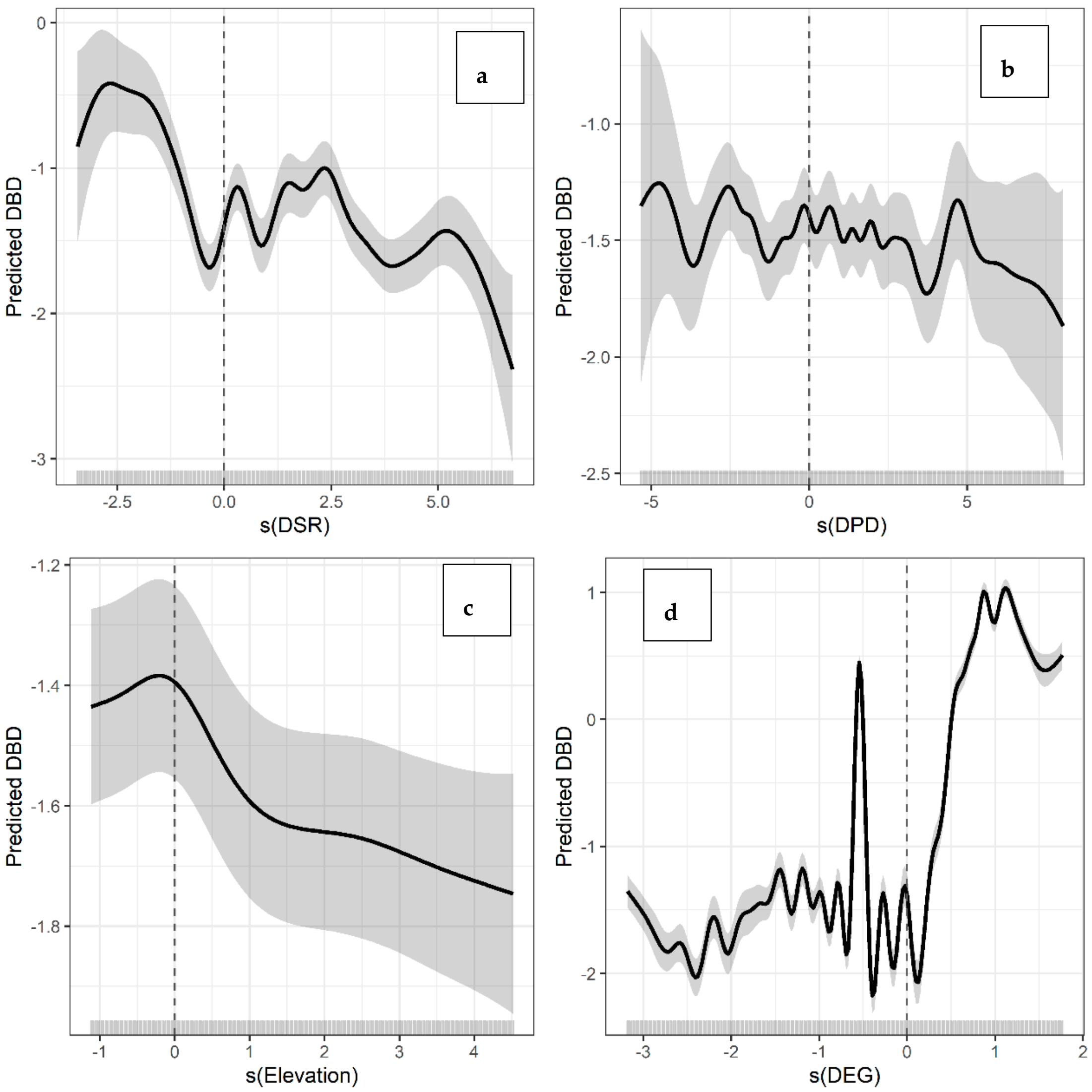

Figure S6. Biplots showing the predicted relationships between β_{sim} change and the four environmental predictors included in the best generalized additive model (GAM) for the BCC 2.6 GCM/RCP combination. (**a**). Change in species richness (ΔSR). (**b**). Change in phylogenetic diversity (ΔPD). (**c**). Change in elevation. (**d**). Change in the average level of ecological generalism (ΔEG). Fitted lines show the univariate GAMs with 95% confidence interval (dark grey). Rugs on the x-axes show the predictor values and how they are distributed. Labels on the y-axes indicate the smooth functions for the term of interest (ΔSR, ΔPD, ΔEG and elevation) and the estimated degrees of freedom (following the term). Values above and below the horizontal dashed line indicate heterogenization and homogenization, respectively. Values left and right of the vertical dashed line indicate in (**a**) species loss and gain, (**b**) PD decrease and increase and (**d**) assemblages composed by specialists and generalists, respectively.

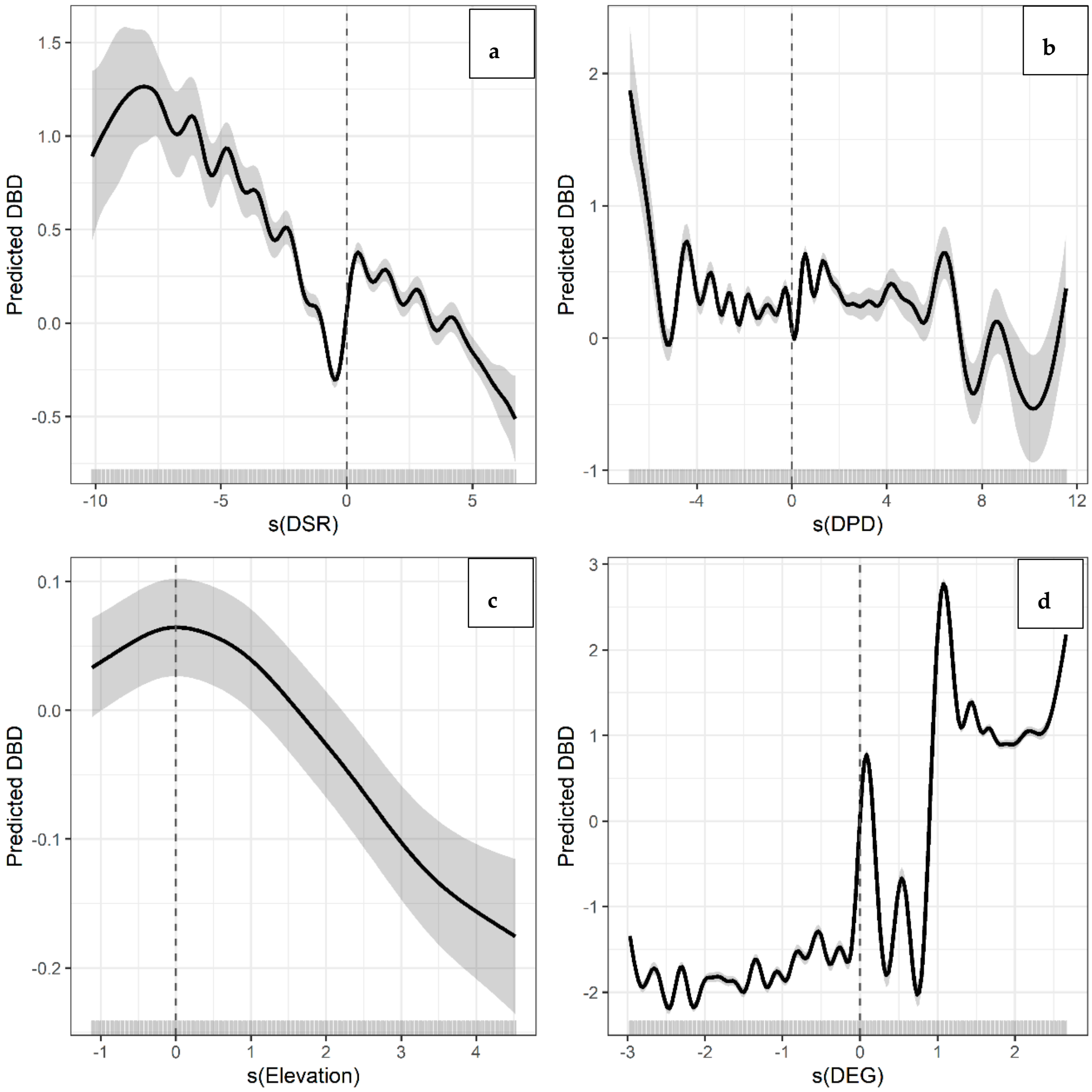

Figure S7. Biplots showing the predicted relationships between β_{sim} change and the four environmental predictors included in the best generalized additive model (GAM) for the BCC 8.5 GCM/RCP combination. (**a**). Change in species richness (ΔSR). (**b**). Change in phylogenetic diversity (ΔPD). (**c**). Change in elevation. (**d**). Change in the average level of ecological generalism (ΔEG). Fitted lines show the univariate GAMs with 95% confidence interval (dark grey). Rugs on the x-axes show the predictor values and how they are distributed. Labels on the y-axes indicate the smooth functions for the term of interest (ΔSR, ΔPD, ΔEG and elevation) and the estimated degrees of freedom (following the term). Values above and below the horizontal dashed line indicate heterogenization and homogenization, respectively. Values left and right of the vertical dashed line indicate in (**a**) species loss and gain, (**b**) PD decrease and increase and (**d**) assemblages composed by specialists and generalists, respectively.

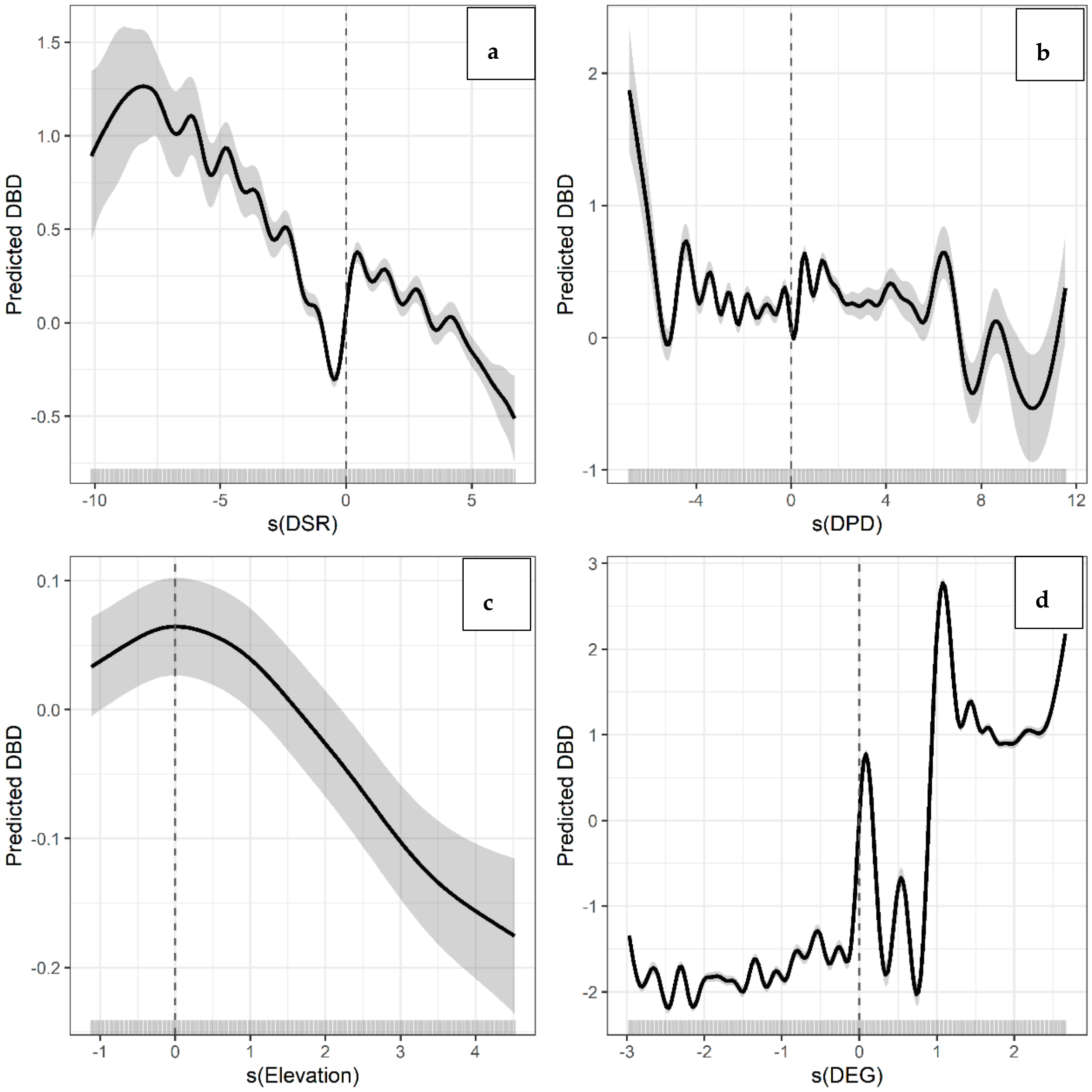

Figure S8. Biplots showing the predicted relationships between β_{sim} change and the four environmental predictors included in the best generalized additive model (GAM) for the CCSM4 8.5 GCM/RCP combination. (**a**). Change in species richness (ΔSR). (**b**). Change in phylogenetic diversity (ΔPD). (**c**). Change in elevation. (**d**). Change in the average level of ecological generalism (ΔEG). Fitted lines show the univariate GAMs with 95% confidence interval (dark grey). Rugs on the x-axes show the predictor values and how they are distributed. Labels on the y-axes indicate the smooth functions for the term of interest (ΔSR, ΔPD, ΔEG and elevation) and the estimated degrees of freedom (following the term). Values above and below the horizontal dashed line indicate heterogenization and homogenization, respectively. Values left and right of the vertical dashed line indicate in (**a**) species loss and gain, (**b**) PD decrease and increase and (**d**) assemblages composed by specialists and generalists, respectively.

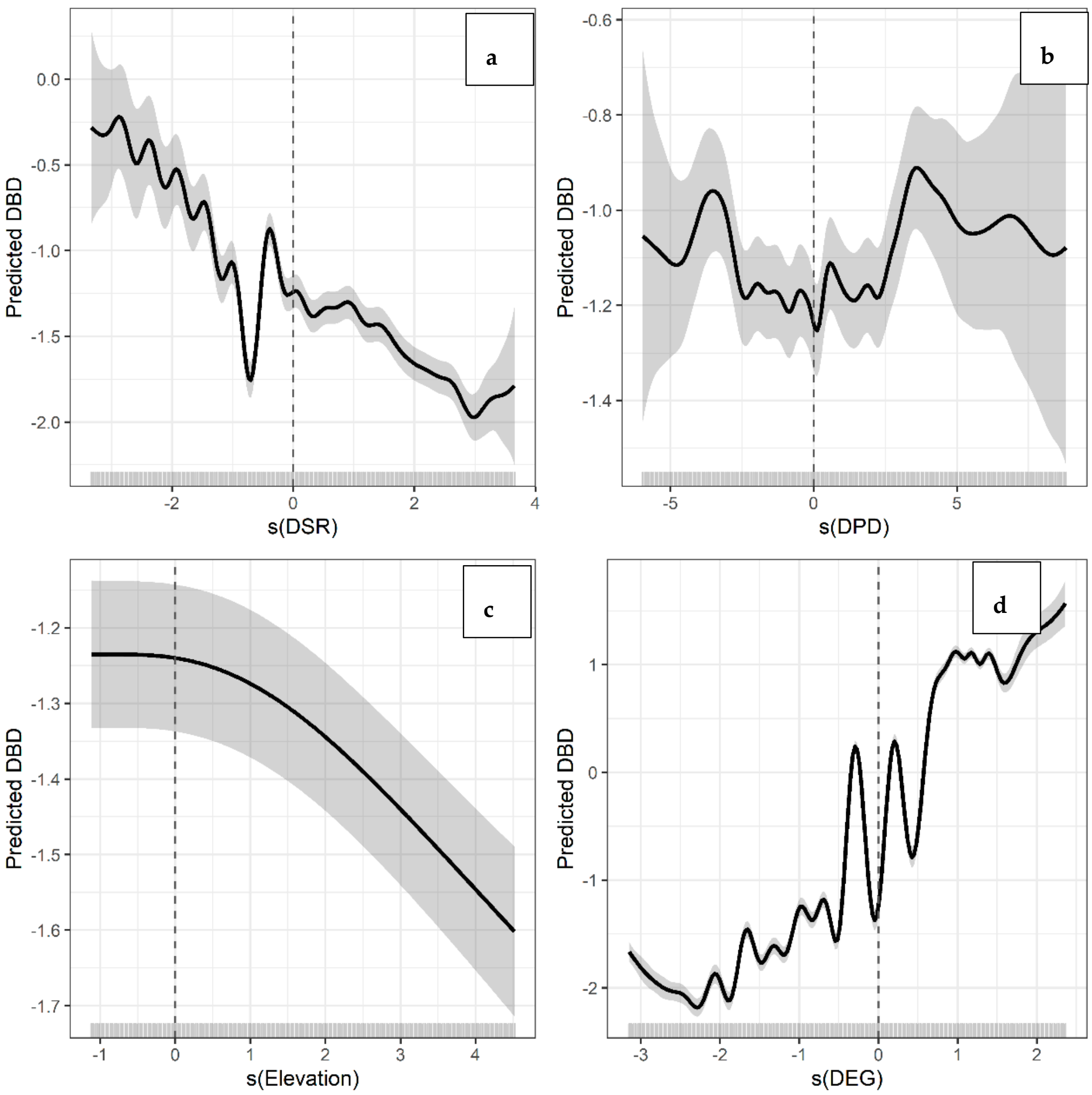

Figure S9. Biplots showing the predicted relationships between β_{sim} change and the four environmental predictors included in the best generalized additive model (GAM) for the HadGEM2 2.6 GCM/RCP combination. (**a**). Change in species richness (ΔSR). (**b**). Change in phylogenetic diversity (ΔPD). (**c**). Change in elevation. (**d**). Change in the average level of ecological generalism (ΔEG). Fitted lines show the univariate GAMs with 95% confidence interval (dark grey). Rugs on the x-axes show the predictor values and how they are distributed. Labels on the y-axes indicate the smooth functions for the term of interest (ΔSR, ΔPD, ΔEG and elevation) and the estimated degrees of freedom (following the term). Values above and below the horizontal dashed line indicate heterogenization and homogenization, respectively. Values left and right of the vertical dashed line indicate in (**a**) species loss and gain, (**b**) PD decrease and increase and (**d**) assemblages composed by specialists and generalists, respectively.

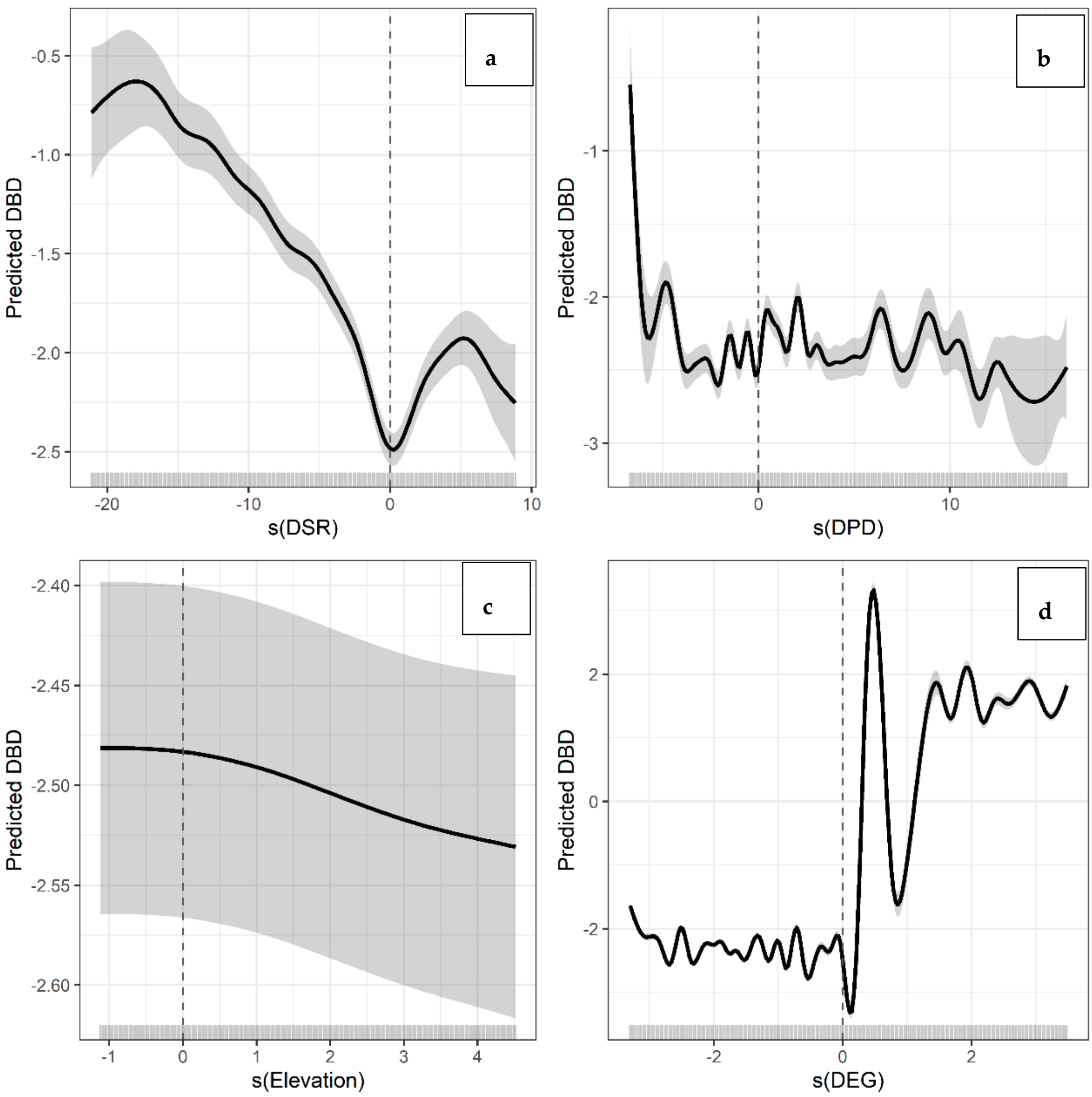

Figure S10. Biplots showing the predicted relationships between β_{sim} change and the four environmental predictors included in the best generalized additive model (GAM) for the HadGEM2 8.5 GCM/RCP combination. (**a**). Change in species richness (ΔSR). (**b**). Change in phylogenetic diversity (ΔPD). (**c**). Change in elevation. (**d**). Change in the average level of ecological generalism (ΔEG). Fitted lines show the univariate GAMs with 95% confidence interval (dark grey). Rugs on the x-axes show the predictor values and how they are distributed. Labels on the y-axes indicate the smooth functions for the term of interest (ΔSR, ΔPD, ΔEG and elevation) and the estimated degrees of freedom (following the term). Values above and below the horizontal dashed line indicate heterogenization and homogenization, respectively. Values left and right of the vertical dashed line indicate in (**a**) species loss and gain, (**b**) PD decrease and increase and (**d**) assemblages composed by specialists and generalists, respectively.

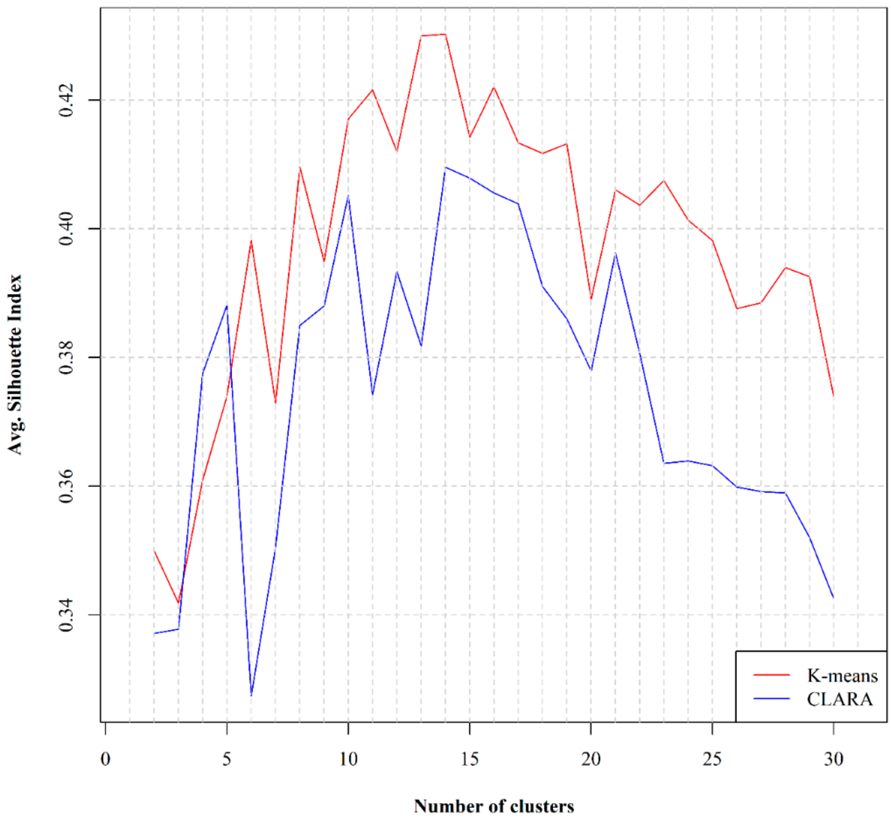

Figure S11. The values of the Silhouette index for the k-means and the CLARA unsupervised clustering algorithms regarding the optimal number of biogeographical sectors (clusters) currently occurring in Crete.

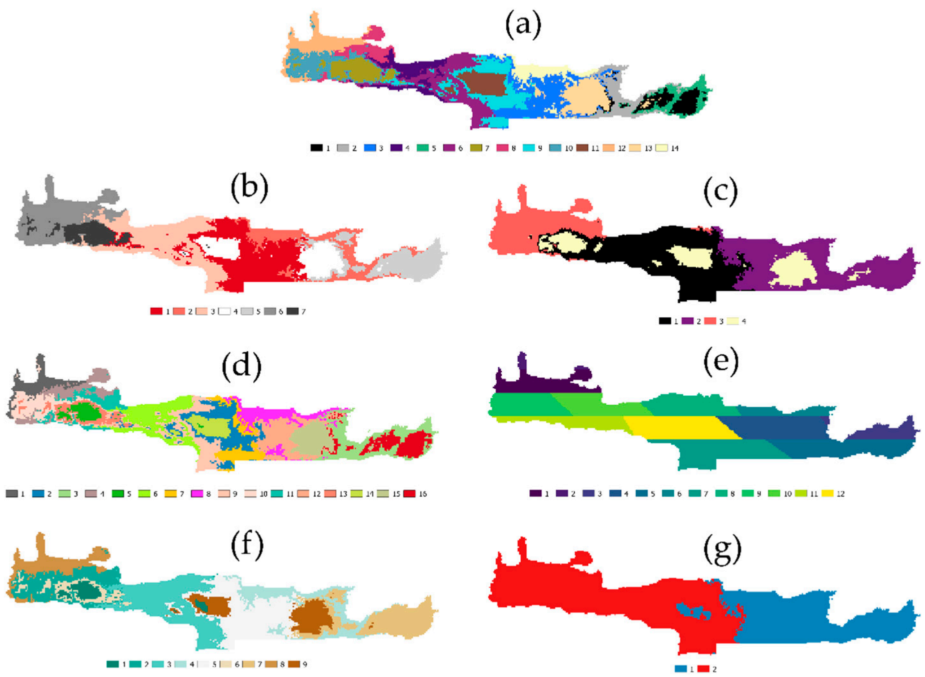

Figure S12. Bioregionalization of Crete for (**a**) the current time-period, (**b**) the BCC Global Circulation Model (GCM) and the Representative Concentration Pathway (RCP) 2.6, (**c**) the BCC 8.5 GCM/RCP, (**d**) the CCSM4 2.6 GCM/RCP, (**e**) the CCSM4 8.5 GCM/RCP, (**f**) the HadGEM2 2.6 GCM/RCP and (**g**) the HadGEM2 8.5 GCM/RCP. Each colour indicates a different biogeographical sector.

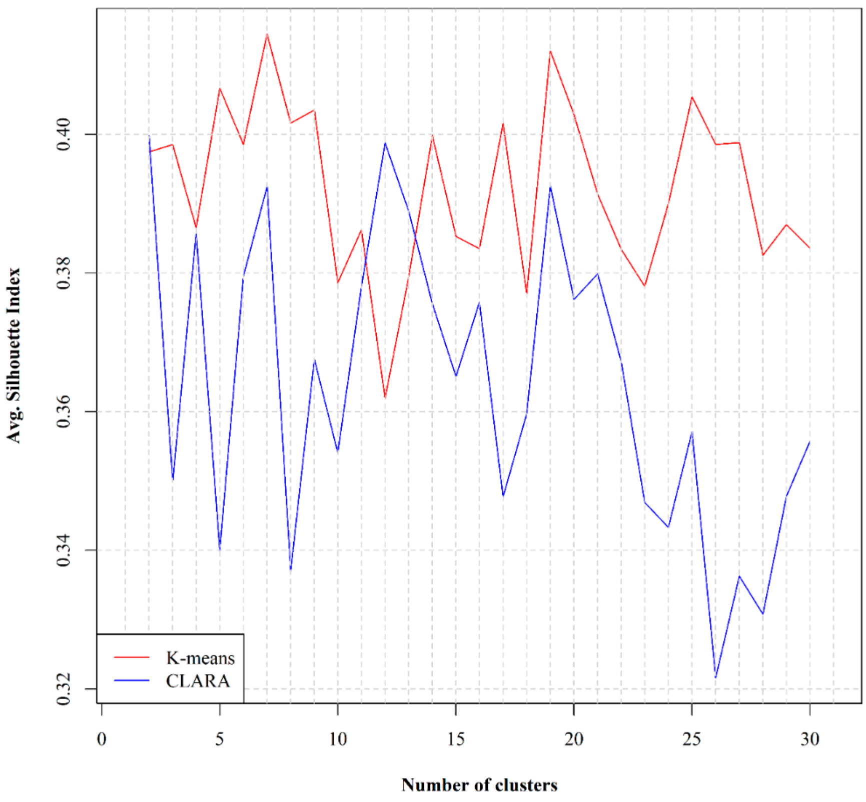

Figure S13. The values of the Silhouette index for the k-means and the CLARA unsupervised clustering algorithms regarding the optimal number of biogeographical sectors (clusters) predicted to occur for the BCC 2.6 GCM/RCP combination in Crete.

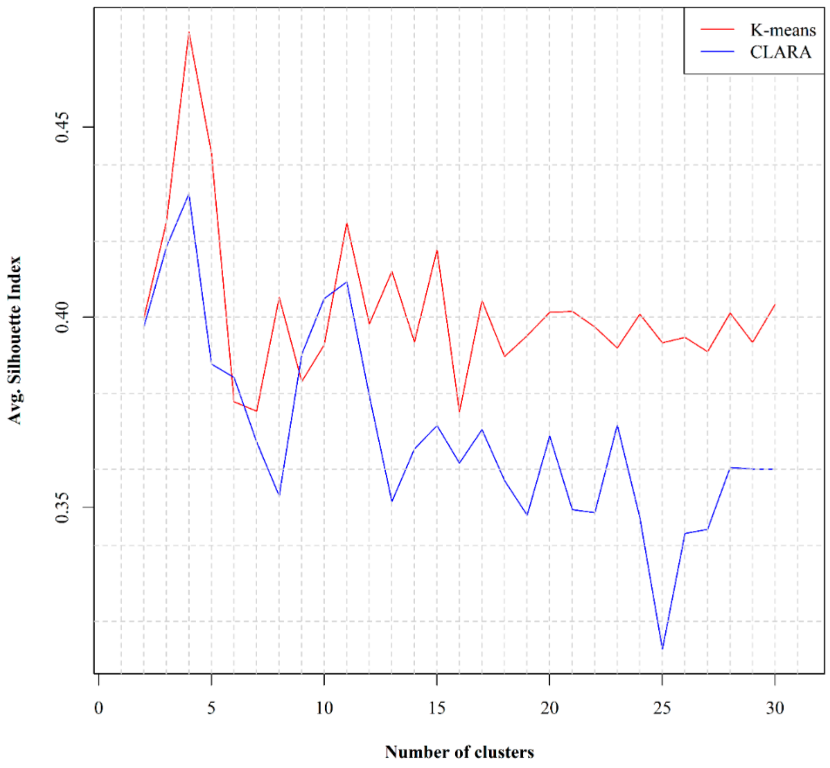

Figure S14. The values of the Silhouette index for the k-means and the CLARA unsupervised clustering algorithms regarding the optimal number of biogeographical sectors (clusters) predicted to occur for the BCC 8.5 GCM/RCP combination in Crete.

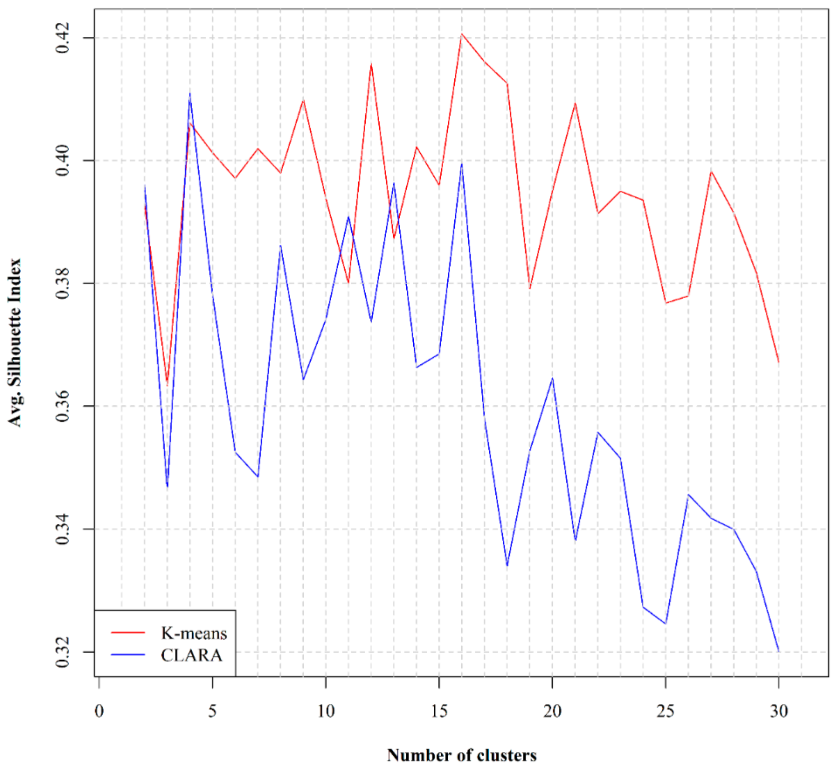

Figure S15. The values of the Silhouette index for the k-means and the CLARA unsupervised clustering algorithms regarding the optimal number of biogeographical sectors (clusters) predicted to occur for the CCSM4 2.6 GCM/RCP combination in Crete.

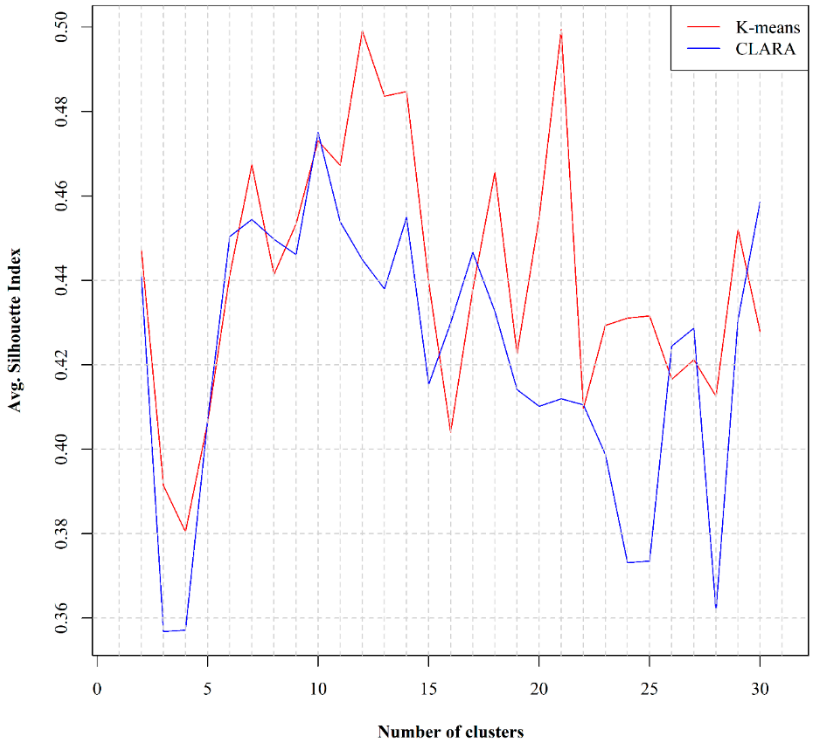

Figure S16. The values of the Silhouette index for the k-means and the CLARA unsupervised clustering algorithms regarding the optimal number of biogeographical sectors (clusters) predicted to occur for the CCSM4 8.5 GCM/RCP combination in Crete.

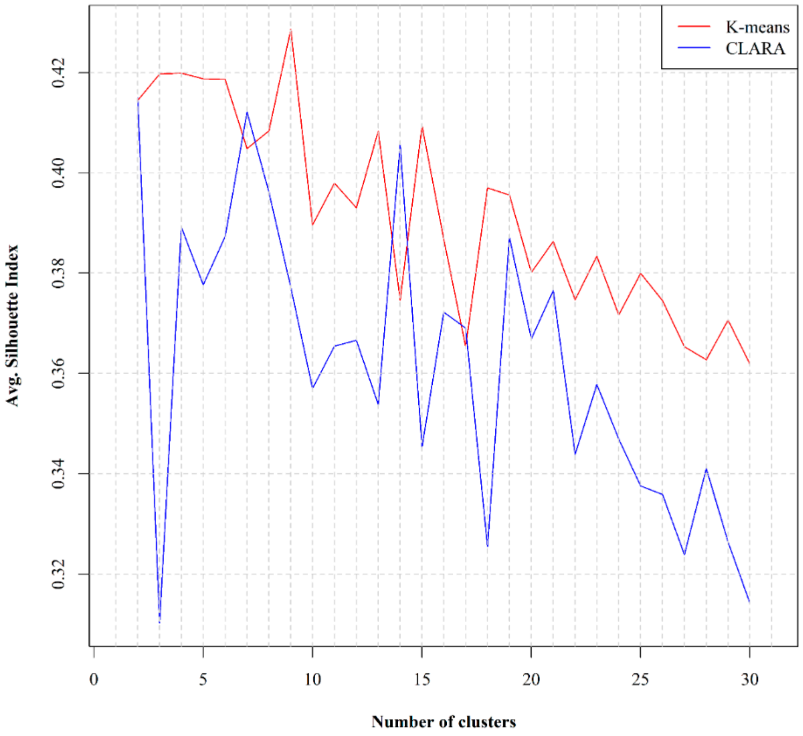

Figure S17. The values of the Silhouette index for the k-means and the CLARA unsupervised clustering algorithms regarding the optimal number of biogeographical sectors (clusters) predicted to occur for the HadGEM2 2.6 GCM/RCP combination in Crete.

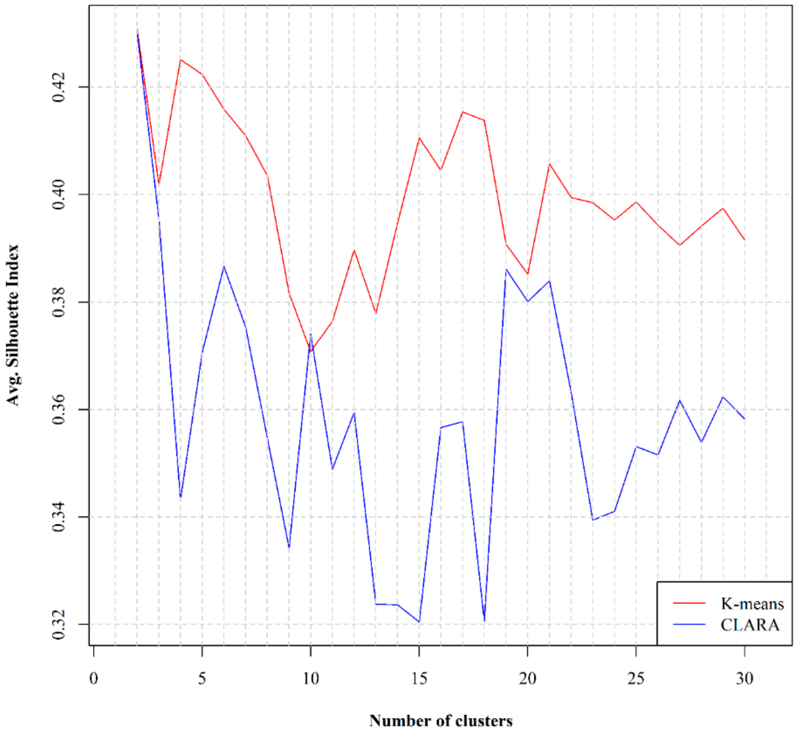

Figure S18. The values of the Silhouette index for the k-means and the CLARA unsupervised clustering algorithms regarding the optimal number of biogeographical sectors (clusters) predicted to occur for the HadGEM2 8.5 GCM/RCP combination in Crete.

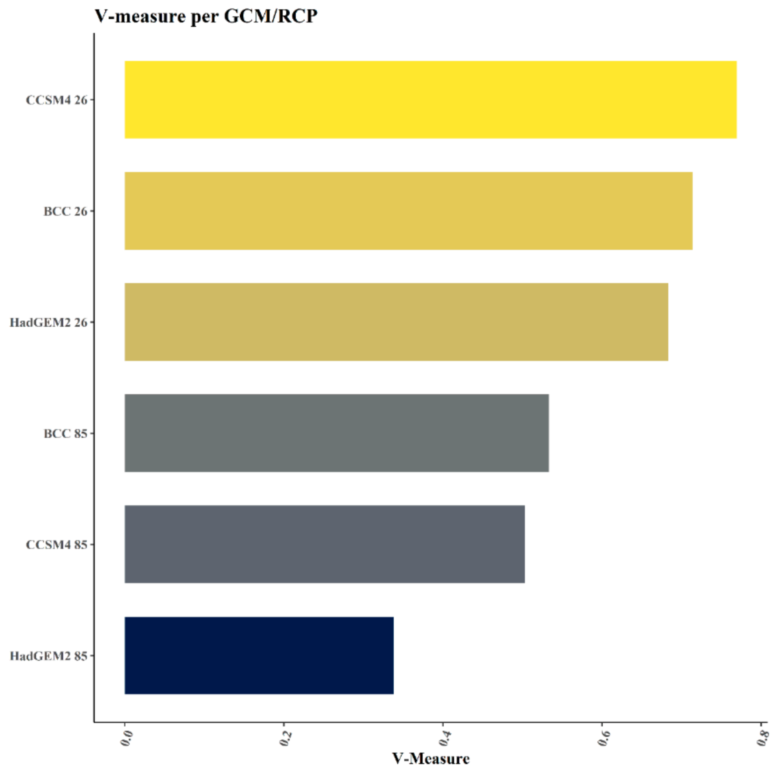

Figure S19. Similarity regarding Crete's bioregionalization schema between the present and each Global Circulation Model (GCM) and Representative Concentration Pathway (RCP), based on the V-measure index.

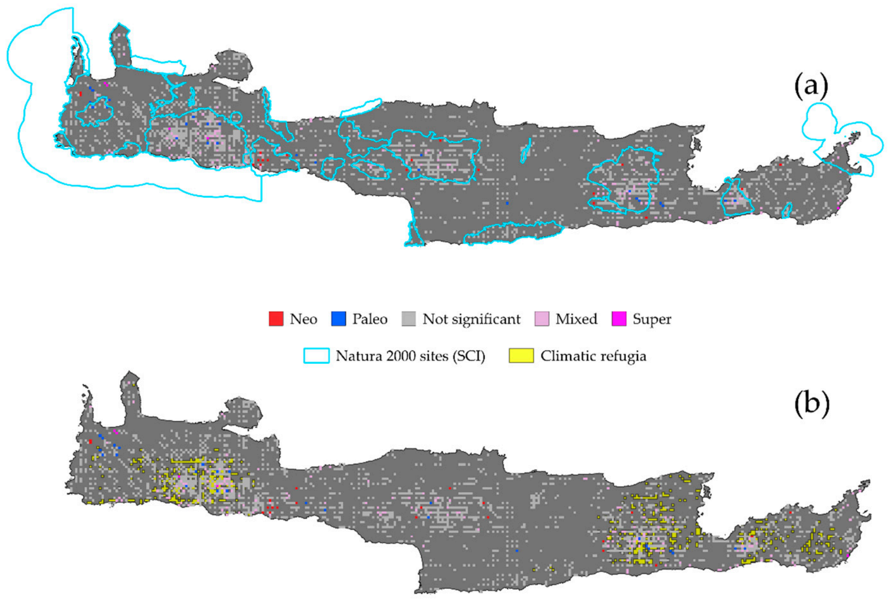

Figure S20. (**a**) Map of the protected areas (PA) network in Crete overlaid onto the Categorical Analysis of Neo- and Paleo-Endemism (CANAPE) results, (**b**) Map of the recognised climate refugia in Crete overlaid onto the CANAPE results. SCI: Sites of Community Importance. Dark grey cells contain no records.

References

1. Faith, D.P. Conservation evaluation and phylogenetic diversity. *Biol. Conserv.* **1992**, doi:10.1016/0006-3207(92)91201-3.
2. Kembel, S.W.; Cowan, P.D.; Helmus, M.R.; Cornwell, W.K.; Morlon, H.; Ackerly, D.D.; Blomberg, S.P.; Webb, C.O. Picante: R tools for integrating phylogenies and ecology. *Bioinformatics* **2010**, doi:10.1093/bioinformatics/btq166.
3. Tsirogiannis, C.; Sandel, B. PhyloMeasures: a package for computing phylogenetic biodiversity measures and their statistical moments. *Ecography* **2016**, *39*, 709–714, doi:10.1111/ecog.01814.
4. Mazel, F.; Renaud, J.; Guilhaumon, F.; Mouillot, D.; Gravel, D.; Thuiller, W. Mammalian phylogenetic diversity-area relationships at a continental scale. *Ecology* **2015**, doi:10.1890/14-1858.1.
5. Laffan, S.W.; Lubarsky, E.; Rosauer, D.F. Biodiverse, a tool for the spatial analysis of biological and related diversity. *Ecography*. **2010**, *33*, 643–647, doi:10.1111/j.1600-0587.2010.06237.x.
6. Mishler, B.D.; Knerr, N.; González-Orozco, C.E.; Thornhill, A.H.; Laffan, S.W.; Miller, J.T. Phylogenetic measures of biodiversity and neo-and paleo-endemism in Australian acacia. *Nat. Commun*. **2014**, *5*, doi:10.1038/ncomms5473.
7. Rosauer, D.; Laffan, S.W.; Crisp, M.D.; Donnellan, S.C.; Cook, L.G. Phylogenetic endemism: A new approach for identifying geographical concentrations of evolutionary history. *Mol. Ecol.* **2009**, doi:10.1111/j.1365-294X.2009.04311.x.
8. Dormann, C.F.; Elith, J.; Bacher, S.; Buchmann, C.; Carl, G.; Carré, G.; Marquéz, J.R.G.; Gruber, B.; Lafourcade, B.; Leitão, P.J.; et al. Collinearity: A review of methods to deal with it and a simulation study evaluating their performance. *Ecography* **2013**, *36*, 27–46, doi:10.1111/j.1600-0587.2012.07348.x.
9. Naimi, B.; Hamm, N.A.S.; Groen, T.A.; Skidmore, A.K.; Toxopeus, A.G. Where is positional uncertainty a problem for species distribution modelling? *Ecography* **2014**, *37*, 191–203, doi:10.1111/j.1600-0587.2013.00205.x.
10. Barton, K. MuMIn: Multi-Model Inference. 2017. Available online: https://CRAN.R-project.org/package=MuMIn (accessed on 20 October 2019)
11. Burnham, K.P.; Anderson, D.R. Model selection and multimodel inference: a practical information-theoretic approach; Springer Science & Business Media: Berlin, Germany, 2003.
12. Cameron, R. a D.; Triantis, K. a.; Parent, C.E.; Guilhaumon, F.; Alonso, M.R.; Ibáñez, M.; de Frias Martins, A.M.; Ladle, R.J.; Whittaker, R.J. Snails on oceanic islands: Testing the general dynamic model of oceanic island biogeography using linear mixed effect models. *J. Biogeogr*. **2013**, *40*, 117–130, doi:10.1111/j.1365-2699.2012.02781.x.
13. Lüdecke, D. sjstats: Statistical Functions for Regression Models 2017. Available online: https://CRAN.R-project.org/package= sjstats (accessed on 20 October 2019)
14. Bruelheide, H.; Dengler, J.; Jiménez-Alfaro, B.; Purschke, O.; Hennekens, S.M.; Chytrý, M.; Pillar, V.D.; Jansen, F.; Kattge, J.; Sandel, B.; et al. sPlot – A new tool for global vegetation analyses. *J. Veg. Sci*. **2019**, *30*, 161–186, doi:10.1111/jvs.12710.
15. Maitner, B.S.; Boyle, B.; Casler, N.; Condit, R.; Donoghue, J.; Durán, S.M.; Guaderrama, D.; Hinchliff, C.E.; Jørgensen, P.M.; Kraft, N.J.B.; et al. The bien r package: A tool to access the Botanical Information and Ecology Network (BIEN) database. *Methods Ecol. Evol.* **2018**, *9*, 373–379, doi:10.1111/2041-210X.12861.
16. Jin, Y.; Qian, H. V.PhyloMaker: an R package that can generate very large phylogenies for vascular plants. *Ecography* **2019**, doi:10.1111/ecog.04434.

© 2020 by the authors. Submitted for possible open access publication under the terms and conditions of the Creative Commons Attribution (CC BY) license (http://creativecommons.org/licenses/by/4.0/).
